# Genetic diversity, population structure, and genome-wide association study for the flowering trait in a diverse panel of 428 moth bean (*Vigna aconitifolia*) accessions using genotyping by sequencing

**DOI:** 10.1186/s12870-023-04215-w

**Published:** 2023-04-29

**Authors:** Arvind Kumar Yadav, Chandan Kumar Singh, Rajwant K. Kalia, Shikha Mittal, Dhammaprakash P. Wankhede, Rajesh K. Kakani, Shraddha Ujjainwal, Aakash, Ankit Saroha, N. S. Nathawat, Reena Rani, Pooja Panchariya, Manoj Choudhary, Kantilal Solanki, K. K. Chaturvedi, Sunil Archak, Kuldeep Singh, Gyanendra Pratap Singh, Amit Kumar Singh

**Affiliations:** 1 ICAR- National Bureau of Plant Genetic Resources, Pusa Campus, New Delhi, Delhi India; 2 ICAR- Central Arid Zone Research Institute, Jodhpur, Rajasthan India; 3 Department of Biotechnology and Bioinformatics, Jaypee University of Information Technology, Solan, Himachal Pradesh India; 4 ICAR- Central Arid Zone Research Institute, Regional Research Station, Bikaner, Rajasthan India; 5 ICAR- Indian Agricultural Statistics Research Institute, New Delhi, Delhi India; 6 International Crops Research Institute for the Semi-Arid Tropics, Hyderabad, Telangana India

**Keywords:** Flowering time, GBS, GWAS, Multi-locus association, Moth bean, SNPs

## Abstract

**Background:**

Moth bean (*Vigna aconitifolia*) is an underutilized, protein-rich legume that is grown in arid and semi-arid areas of south Asia and is highly resistant to abiotic stresses such as heat and drought. Despite its economic importance, the crop remains unexplored at the genomic level for genetic diversity and trait mapping studies. To date, there is no report of SNP marker discovery and association mapping of any trait in this crop. Therefore, this study aimed to dissect the genetic diversity, population structure and marker-trait association for the flowering trait in a diversity panel of 428 moth bean accessions using genotyping by sequencing (GBS) approach.

**Results:**

A total of 9078 high-quality single nucleotide polymorphisms (SNPs) were discovered by genotyping of 428 moth bean accessions. Model-based structure analysis and PCA grouped the moth bean accessions into two subpopulations. Cluster analysis revealed accessions belonging to the Northwestern region of India had higher variability than accessions from the other regions suggesting that this region represents its center of diversity. AMOVA revealed more variations within individuals (74%) and among the individuals (24%) than among the populations (2%). Marker-trait association analysis using seven multi-locus models including mrMLM, FASTmrEMMA FASTmrEMMA, ISIS EM-BLASSO, MLMM, BLINK and FarmCPU revealed 29 potential genomic regions for the trait days to 50% flowering, which were consistently detected in three or more models. Analysis of the allelic effect of the major genomic regions explaining phenotypic variance of more than 10% and those detected in at least 2 environments showed 4 genomic regions with significant phenotypic effect on this trait. Further, we also analyzed genetic relationships among the *Vigna* species using SNP markers. The genomic localization of moth bean SNPs on genomes of closely related *Vigna* species demonstrated that maximum numbers of SNPs were getting localized on *Vigna mungo.* This suggested that the moth bean is most closely related to *V. mungo.*

**Conclusion:**

Our study shows that the north-western regions of India represent the center of diversity of the moth bean. Further, the study revealed flowering-related genomic regions/candidate genes which can be potentially exploited in breeding programs to develop early-maturity moth bean varieties.

**Supplementary Information:**

The online version contains supplementary material available at 10.1186/s12870-023-04215-w.

## Background

Legumes are the basic source of plant protein that replenish the nutrient requirement of a large population, dwelling in underdeveloped and developing countries. Over the years, worldwide demand of pulses has increased manifold due to ever increasing population [1]. Although conventional legume crops cover a major part of required food legumes, the development as well as cultivation of underutilized legume crops such as moth bean [*V. aconitifolia* (Jacq.) Marechal], zombie pea (*Vigna vexillata* (L.) A. Rich), riceban [*Vigna umbellata* (Thunb.) Ohwi & H. Ohashi] which are rich in protein as well as other nutrients. These can serve as an alternative source to counter the demand–supply imbalance of conventional legume crops [2].

Moth bean (*V. aconitifolia*) is a heat and drought-tolerant legume, generally grown in arid and semi-arid regions of Asian countries including India [3]. It belongs to the family *Fabaceae,* subfamily *Papilionaceae*, genus *Vigna*, subgenus *Ceratotropis* and section *Aconitifoliae* [3]. Moth bean is popularly known as matki, mat bean, or dew bean. Its protein-rich dried-seed are used as ‘Dal’ and other preparations and immature green pods as a vegetable [4]. Moth bean shows high resilience towards drought stress when compared to all other *Kharif* pulses. It also fulfills the nutritional need of a large population dwelling in developing counties [5]. The growing condition can reach the maximum temperature of 45 °C with an annual rainfall of 200–300 mm. Its seeds are highly nutritious and contain approximately 22–24% protein with essential amino acids such as lysine and leucine [6, 7]. Moth bean also helps in decreasing soil erosion, soil temperature and improves soil nutrients through nitrogen fixation [6]. With these qualities, the moth bean is considered as a future legume crop for developing and underdeveloped countries that have already been severely impacted by climate change.

In recent years genome-wide association study (GWAS) has become an efficient method to study the association of specific traits with molecular polymorphism [8]. Furthermore, the availability of low-cost genotyping techniques such as genotyping by sequencing (GBS) has helped the identification of large number of single nucleotide polymorphisms (SNPs). The GBS approach provides opportunity to perform GWAS for the desirable traits in many crops including those without the reference genome [9–11]. GWAS using high-density molecular markers and a diverse genotypic population provides a high-resolution genomic map than conventional quantitative trait locus (QTL) mapping. This approach has been used to examine the genetic basis of complex traits in model legume crop *Medicago truncatula* [12–14] and many other legume crops such as soybean [15–17], chickpea [18–21], cowpea [22–24], pigeon pea [25] and mung bean [26]. Various traits such as seed size, seed weight, flowering time, and the number of pods are considered important as these can help in breeding high-yielding varieties to improve the overall production of legume crops. Amongst these significant traits, floral timing is of great importance for crop cultivation as it significantly influences agronomic traits like plant height, plant growth, and grain quality [27, 28]. Therefore, flowering time has a considerable impact on the economic yield of crops. However, flowering is a complex trait controlled by internal and external factors [29]. Larger variation in the flowering time of legume crops has added to their improvement via selection and breeding [28]. In many legumes, flowering is stimulated by variation in day length [23]. The flowering transition phenomenon and genetic signaling pathways for flowering have been well studied in the model plant *Arabidopsis* [28, 30]. A total of 306 genes have been characterized in *Arabidopsis* that regulate floral development [31].

Despite excellent nutritional profile of the moth bean, no association study has been reported to understand the genetic variability and architecture of desirable traits. The nonavailability of sufficient genomic resources has limited genetic diversity and gene aping studies in this crop. Bhadkaria et al. [32] recently used 10 ISSRs to deduce the molecular diversity of 25-moth bean accessions. Only two polymorphic markers were observed that clustered moth bean accession based on their geographical location. Limited use of the SSR marker system to dissect the genetics of domestication-related traits in the F_2_ mapping population in moth bean has been reported by Yundaeng et al. [7]. SSRs used in this study were developed from different *Vigna* species such as azuki bean, mung bean, yardlong bean and common bean. Of which, SSRs developed from azuki bean showed the highest amplification rate of 76.2% when screened for polymorphism followed by common bean (72.3%), mung bean (31.8%) and yardlong bean (13.6%) [7]. Further, SSRs were used in the construction of linkage maps of *Vigna* species such as mung bean [33], azuki bean [34], rice bean [35] and yardlong bean [36]. The study also reported a high degree of genomic synteny between moth bean and other *Vigna spp* as common markers were found on the same linkage group with the same or similar order.

The National Genebank (NGB) of India, located at ICAR-National Bureau of Plant Genetic Resources (ICAR-NBPGR) maintains the largest collection of moth bean germplasms (1565 accessions) in the world. This collection is a valuable genetic resource for trait discovery and gene mapping studies. However, the extent and pattern of diversity of this collection are unknown at the genomic level limiting its utilization in breeding programs. In the present study, GBS-derived SNP markers were used to get a snapshot of the genetic diversity and population structure in the subset of the moth bean collection (428 accessions). Further, GWAS was also performed on this moth bean subset to identify candidate genes/genomic regions that potentially regulate the trait days to 50% flowering. Thus, the present study provides novel insights into the genetic basis of flowering trait and identifies trait-specific novel genomic regions/genes. The identified genes may be further characterized using functional genomic approaches and exploited in moth bean breeding programs to develop early maturity varieties.

## Results

### Phenotypic analysis

**Fig. 1:**
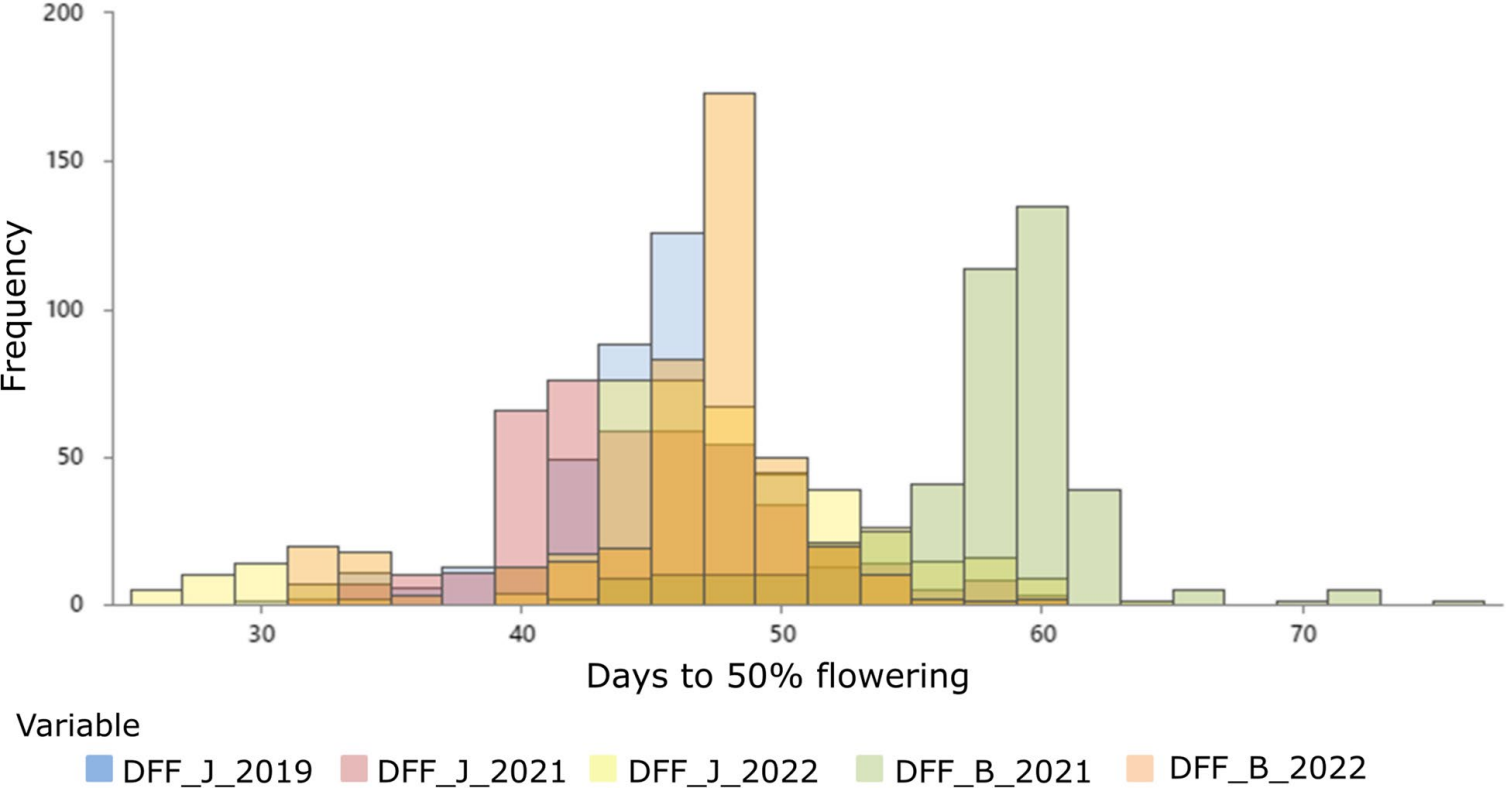
Frequency distribution histogram of phenotypic data for 428 accessions regarding days to 50% flowering (DFF) trait in the moth bean across five datasets. (DFF_J: Days to 50% flowering_Jodhpur; DFF_B: Days to 50% flowring_Bikaner)

The average, median, standard deviation and range for days to 50% flowering were calculated for all 5 environments over two locations. The average days to 50% flowering were 56.99 and 45.62 days at Bikaner in the years 2021 and 2022, respectively. It was 59.47, 44.46, and 46.40 days at Jodhpur in the years 2019, 2021, and 2022, respectively. The maximum number of days to 50% flowering at Bikaner was observed in the year 2021 (75 days) and at Jodhpur in the year 2019 (71 days) (Table 1). The ranges for days to 50% flowering at the Bikaner were 41- 75 and 31- 60 days in the years 2021 and 2022, respectively. The range for days to 50% flowering for the Jodhpur location during 2019, 2021, and 2022 were 31- 71, 31- 60, and 26- 63 respectively. The maximum range for the number of days to 50% flowering was observed in Jodhpur, 2019. The higher values for median and standard deviation were also observed in Jodhpur, 2019. The range of median and standard deviation values was 44 to 61 and 4.66 to 8.92, respectively among the datasets (Table 1). Out of 428 accessions, 26 common accessions showed early flowering across five different environments. These accessions exhibited an average range of 32–38 days for the trait 50% days to flowering (Supplementary Table S1). The overall analysis of all five different data sets showed significant variation for days to 50% flowering trait. Furthermore, the 50% flowering for most of the genotypes was observed in the median stage (Fig. 1).

**Table 1:** Statistical analysis for days to 50% flowering data from different locations

	**Average**	**Median**	**Standard deviation**	**Maximum**	**Minimum**
**Bikaner 2021**	56.99	58	4.66	75	41
**Bikaner 2022**	45.62	47	5.15	60	31
**Jodhpur 2019**	59.47	61	8.92	71	31
**Jodhpur 2021**	44.46	44	5.08	60	31
**Jodhpur 2022**	46.40	47	6.97	63	26

### SNPs identification by GBS method

A total of 200.12 GB data was obtained by the sequencing of GBS libraries of 428 moth bean accessions. The GBS raw data was subject to SNP calling and normalization processes. A total of 9078 high-quality SNPs were used after applying filters such as read depth, missing data, and minor allele frequency (MAF). Filtered SNPs were further divided into six types of SNPs i.e. [A/C], [A/G], [A/T], [C/G], [C/T] and [G/T] having a share of 7.89%, 36.15%, 6.54%, 5.78%, 36.20%, and 7.4% of the total SNPs, respectively. Amongst these, SNP types [A/G] and [C/T] shared the highest percentage while other types contained less than 10% of total SNPs.

### Genetic structure and phylogenic analysis

**Fig. 2:**
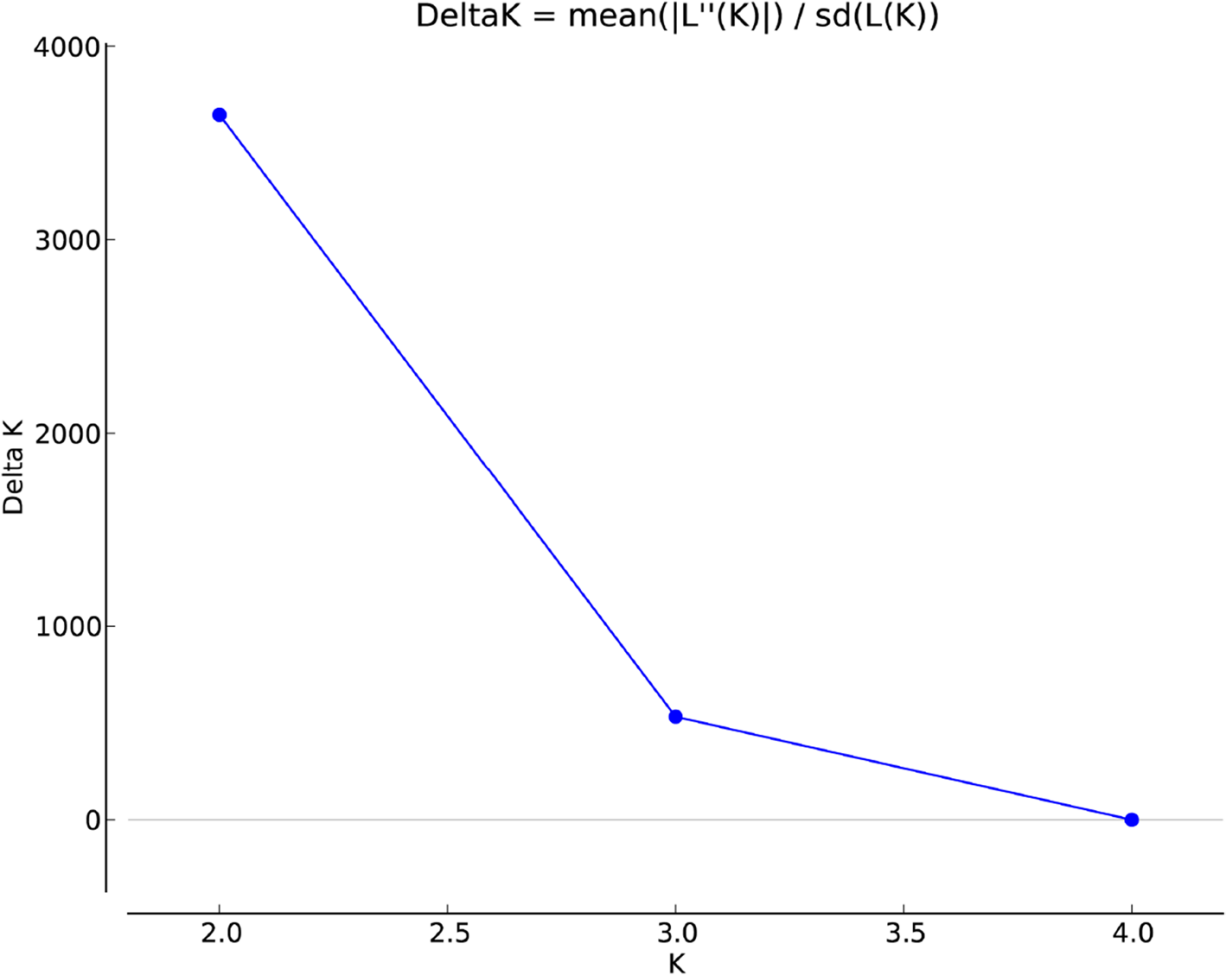
Plot showing the delta K value on the y-axis and the corresponding K values on the x-axis. The delta K peak corresponds to K = 2

**Fig. 3:**
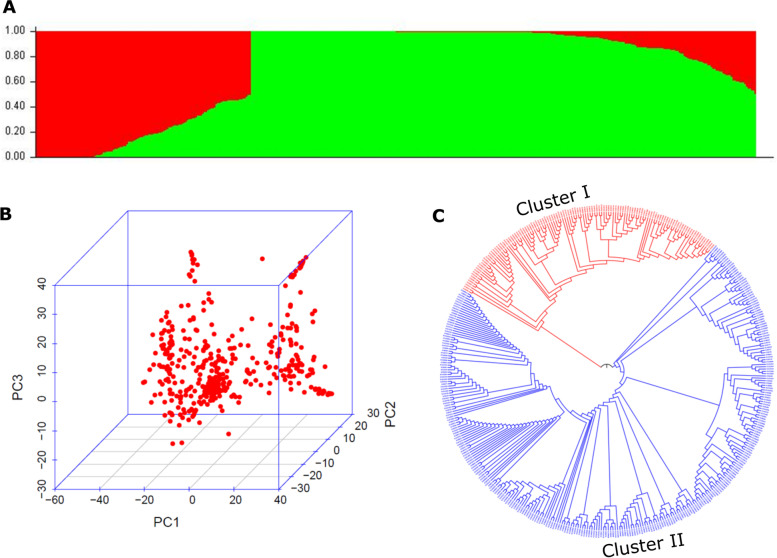
**A** Population structure of moth bean accessions showing two subpopulations, where the red color corresponds to subpopulation 1 and the green color to subpopulation 2. **B** Principal component analysis plot showing 428 accessions into two subpopulations. **C** Phylogenetic tree of 428 accessions using NJ method, where the red color corresponds to cluster I and the blue one to cluster II

To understand the population structure of the moth bean diversity panel, 9078 SNPs were employed in STRUCTURE software using an admixture-based model. The maximum peak value for Delta K was observed at K = 2 (Fig. 2) suggesting 2 subpopulations in the moth bean diversity panel (Fig. 3A). The accessions of the two subpopulations were categorized as pure or admixture based on the level of genetic similarity among individuals of each subpopulations. Individuals with a similarity level ≥ 0.80 were considered as pure and others as admixture. A total of 63 accessions in subpopulation 1 and 254 accessions in subpopulation 2 were pure and the remaining 111 accessions of the two subpopulations were categorized as admixtures (Supplementary Table S2). Principal component analysis (PCA) further depicted that all selected genotypes were grouped into two clusters (Fig. 3B). Similarly, the phylogenetic tree drawn using the NJ method also showed that 428 accessions were grouped into two clusters (Fig. 3C). In phylogenetic analysis, a total of 120 and 308 individuals were presented in cluster I and cluster II respectively. Out of 63 pure individuals in subpopulation 1, 60 were common with individuals in cluster I of phylogenetic analysis and out of 254 pure individuals in subpopulation II, 250 individuals were common with individuals in cluster II of phylogenetic analysis. In PCA also individuals were grouped into two clusters. Therefore, broadly, PCA and phylogenetic results were in agreement with the result of admixture-based model that represented the moth bean genotypes were grouped into two clusters. Further, the distribution of genotypes based on the geographical distribution showed that genotypes from Rajasthan were largely confined to subpopulation II (104 accessions) or cluster II (126 accessions) in phylogenetic analysis. This showed the genotypes grouping was mostly according to their geographical distribution.

### Analysis of Molecular Variance (AMOVA) and Principal Coordinates Analysis (PCoA)

**Fig. 4:**
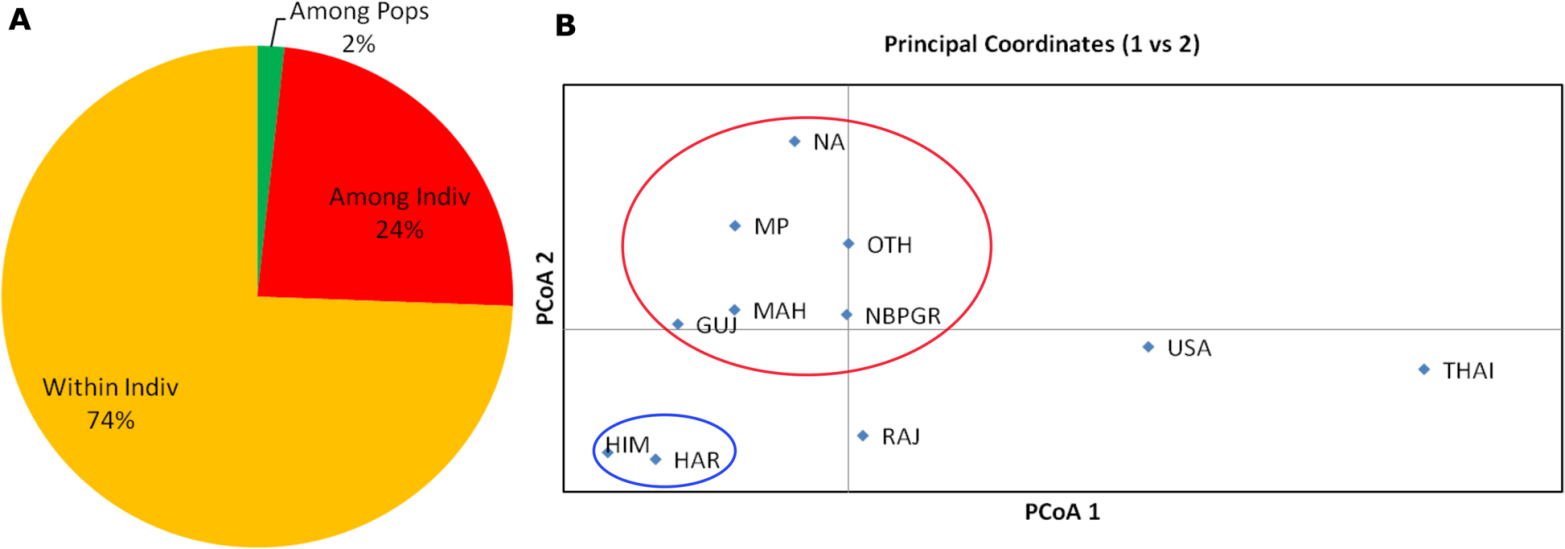
Molecular variability within 428 accessions using 9078 SNP markers. **A** Analysis of Molecular Variance (AMOVA) showing the percentage of molecular variance present among individuals, within individuals and among populations. **B** Principal Coordinates Analysis (PCoA) plot showing genetic variability of collected accessions. Genotypes from Rajasthan are well isolated on the PCoA plot and show a distinct genetic variability. (Pops: Populations; Indiv: Individuals; MP: Madhya Pradesh; MAH: Maharashtra; GUJ: Gujarat; HIM: Himachal Pradesh; HAR: Haryana; RAJ: Rajasthan: NBPGR: National Bureau of Plant Genetic Resources; USA: United States America; THAI: Thailand; OTH: Others; NA: Not Available)

Allelic Distance Matrix for F-Statistics (Fst) analysis in the present study showed a larger variation within the individuals (74%) than among the individual (24%) (Fig. 4A). However, the least variation was observed within the population (2%). Pairwise population Fst Values (FstP) showed the highest genetic differentiation between genotypes originated/collected from Himachal and Haryana with those originated/collected from Thailand and the United States of America (USA). Genotypes originated/collected from Rajasthan were well isolated on principal coordinates analysis (PCoA) plot that showed quite a distinct genetic variability from those collected from other regions (Fig. 4B).

### Localization of moth bean SNPs on the genomes of related *Vigna* species

**Fig. 5:**
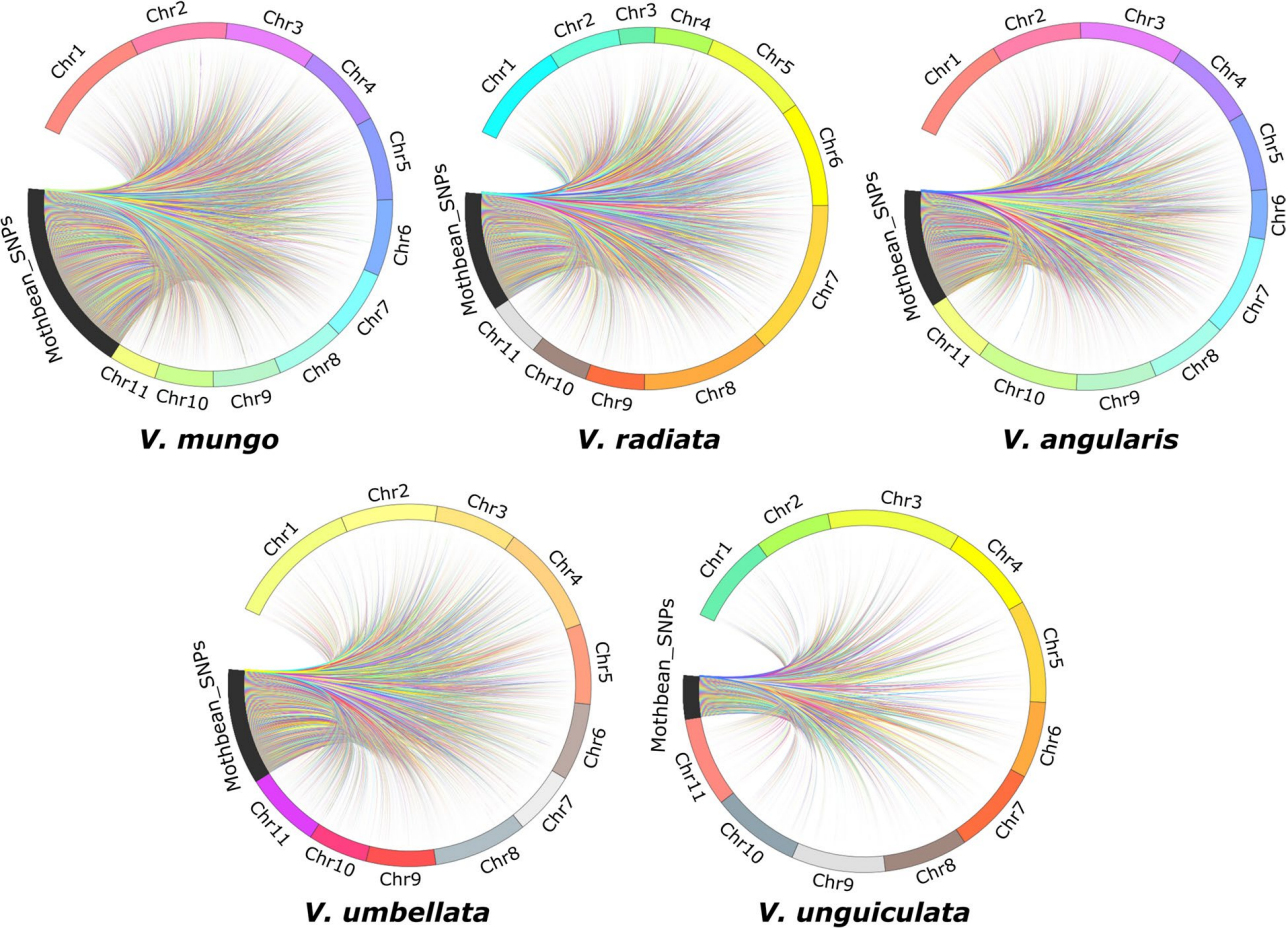
Circular visualization (CIRCOS) plots representing the localization of moth bean SNPs on the chromosomes of other *Vigna* species. The localization is performed by using the 100 bp flanking sequences for all SNPs. The maximum number of SNPs is localized on *V. mungo* followed by *V. radiata* and *V. angularis* and the lowest is on *V. unguiculata*

Our study, reports SNP variants in the moth bean genome for the first time. The chromosomal locations for these SNP could not be assigned due to the non-availability of moth bean reference genome. However, to have a fair idea of their tentative physical location and chromosomal distribution, these can be localized on the genomes of other *Vigna* Species, as they share the same chromosome number (*n* = 10) and are ancestrally related. Further, localization of these SNP on other *Vigna* species genomes was expected to shed some light on the relatedness of moth bean with other *Vigna* species at the DNA level. Therefore, chromosomal localization of significant SNPs (9078) was performed on the genomes of various *Vigna* species such as *V. angularis*, *V. mungo*, *V. radiata*, *V. umbellata*, *V. unguiculata.* BLASTn analysis was performed by using the 100 bp flanking sequences for 9078 SNPs to identify the distribution on *Vigna* genomes. A total of 3721 (40.99%), 6155 (76.80%), 3807 (41.94%), 3681 (40.55%), and 1391 (15.32%) of moth bean SNPs were mapped to the genomes of *V. angularis*, *V. mungo*, *V. radiata*, *V. umbellata*, and *V. unguiculata,* respectively. Based on these sequence-based mapping analyses, the moth bean was found to be closer to *V. mungo* and least close to *V. unguiculata* (Fig. 5, Supplementary Table S3). The other three *Vigna* species, *V. angularis*, *V. radiata*, and *V. umbellata* showed *a* similar level of relationships with moth bean at the DNA level. All moth bean SNPs were evenly distributed across the chromosomes of closely related *Vigna* genomes. The chromosome-wise distribution of moth bean SNPs on closely related *V. mungo* was 11.70%, 8.85%, 7.73%, 8.19%, 9.24%, 6.11%, 9.50%, 9.41%, 12.02%, 8.85%, and 8.38% on the chromosome 1 to 11, respectively. The maximum number of moth bean SNPs were mapped on chromosome 9 (12.02%, a total of 740 SNPs) followed by chromosome 1 (11.70%, a total of 720 SNPs). Further, to get the tentative physical location (order and position on chromosome) of the moth bean SNPs, the chromosomes of *V. mungo* which was found to be most closely related to the moth bean were used as an anchor. These have been depicted in Supplementary Figure S1. In the case of chromosomes 1 and 9, the SNPs were distributed throughout the chromosome. A pool of SNPs was located at a near 18 Mb position on chromosome 9 (Supplementary Figure S1).

### Genome-wide association analysis

**Fig. 6:**
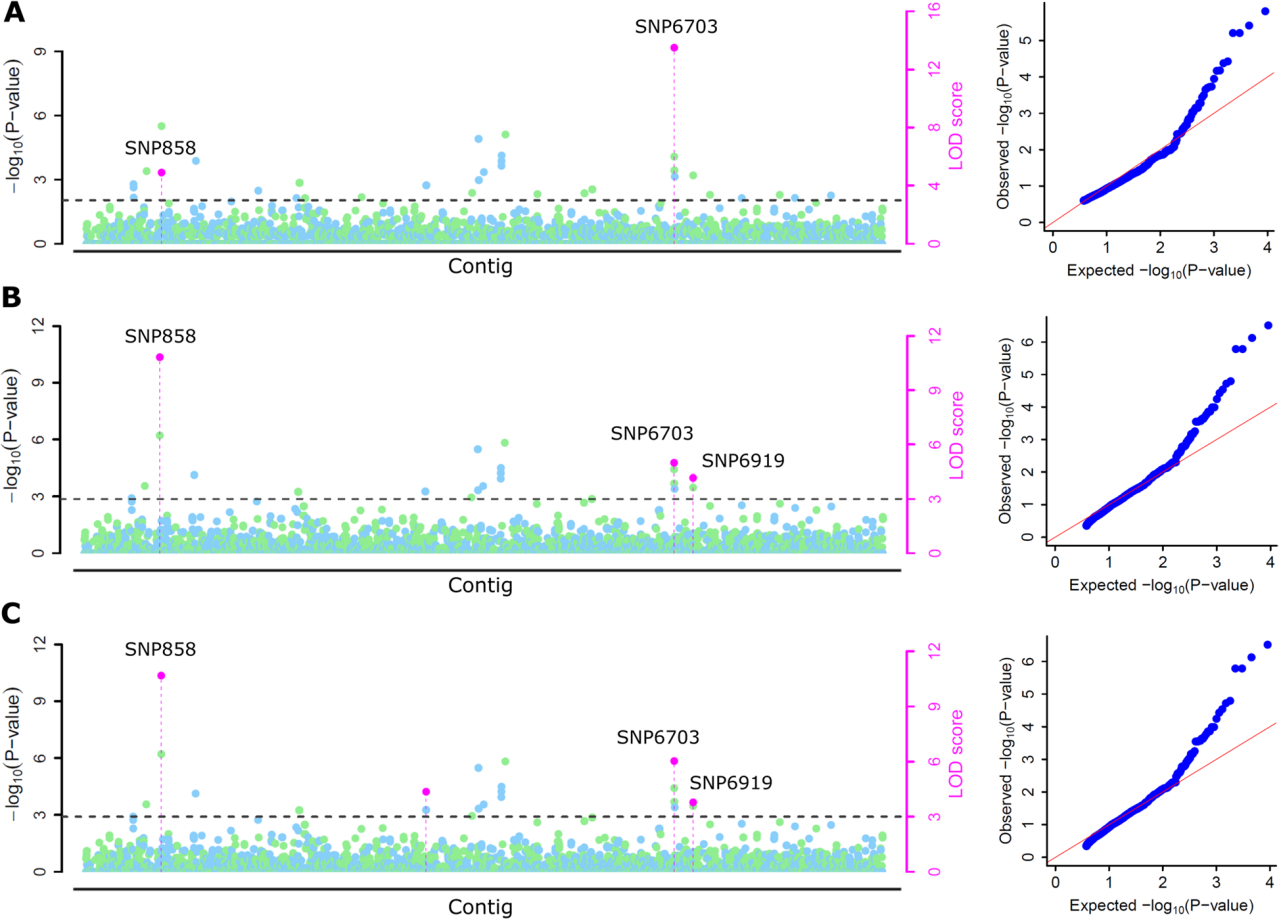
Manhattan and corresponding QQ plots for the association study of flowering trait (Location: Bikaner 2022) using multi-locus models **A** FASTmrEMMA, **B** FASTmrMLM, and **C** mrMLM. In manhattan plots, the horizontal dot line shows the threshold at LOD score of 3. The dots above the threshold line represent significant SNPs. SNPs identified by more than one model are represented by their SNP id. The x-axis shows the location of SNPs on the contigs assembled using GBS data of the moth bean

**Fig. 7:**
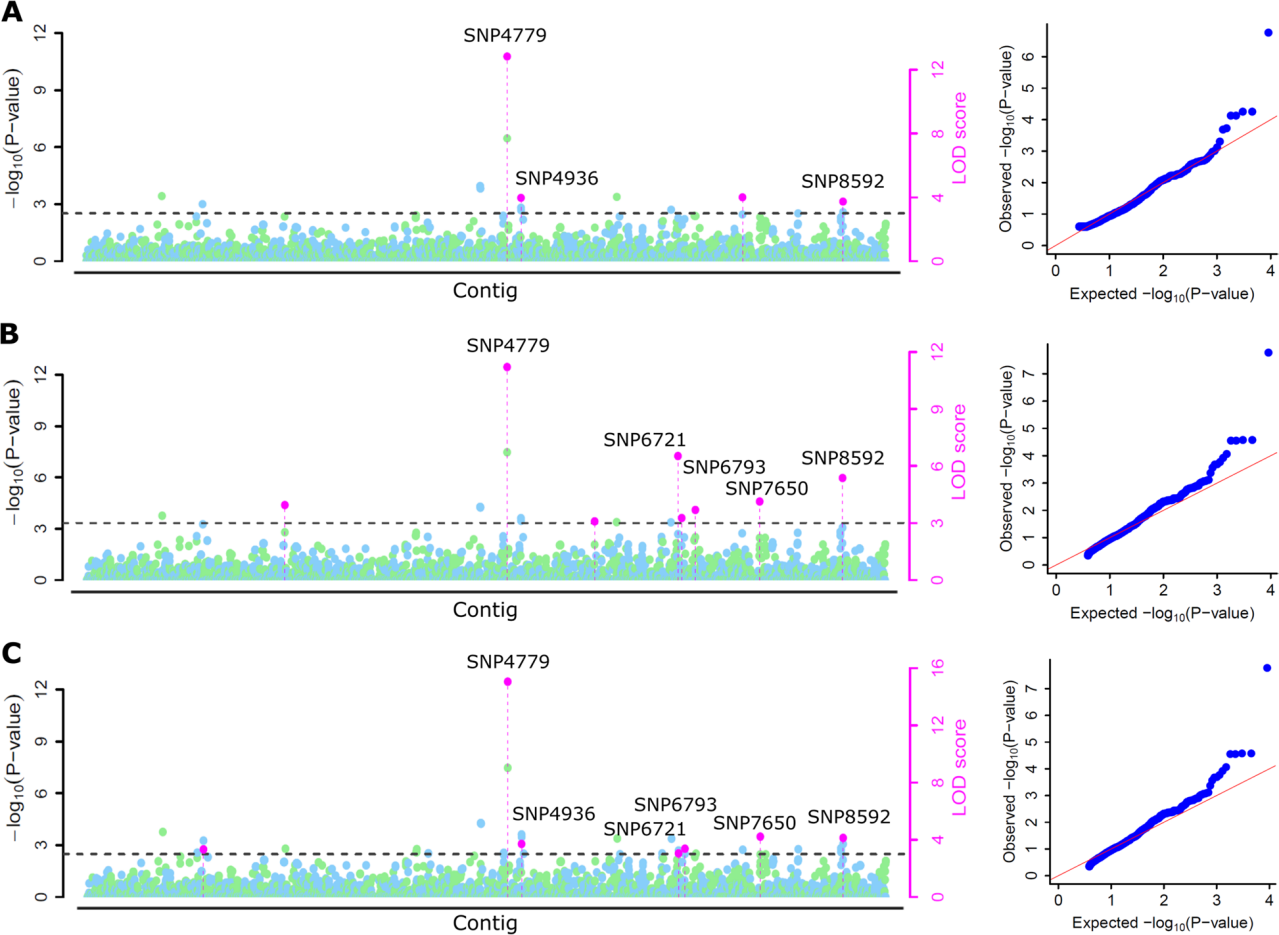
Manhattan and corresponding QQ plots for the association study of flowering trait (Location: Jodhpur 2022) using multi-locus models **A** FASTmrEMMA, **B** FASTmrMLM, and **C** mrMLM. In manhattan plots, the horizontal dot line shows the threshold at LOD score of 3. The dots above the threshold line represent significant SNPs. SNPs identified by more than one model are represented by their SNP id. The x-axis shows the location of SNPs on the contigs assembled using GBS data of the moth bean

The days to 50% flowering phenotyping data of the moth bean diversity panel across the five environments and 9078 SNPs identified through GBS were used in GWAS analysis. GWAS was implemented using various multi-locus models, 4 models (mrMLM, FASTmrEMMA FASTmrEMMA and ISIS EM-BLASSO) in the mrMLM package and 3 models (MLMM, FarmCPU and BLINK) in GAPIT software package. A high significant cut-off logarithm of odds (LOD ≥ 3.0) (Figs. 6 and 7) was given to remove the errors generated due to false associations. A total of 39, 32, 40, 22, and 36 significant SNPs from Bikaner 2021, Bikaner 2022, Jodhpur 2019, Jodhpur 2021, and Jodhpur 2022 were detected using seven multi-locus GWAS methods (Supplementary Table S4). Finally, a total of 34 significant SNPs/genomic regions that were predicted by three or more methods were selected as potential genomic regions for flowering trait across the environments (Table 2). Out of total 34 significant SNPs, 6, 6, 9, 6, and 7 SNPs were identified from Bikaner 2021, Bikaner 2022, Jodhpur 2019, Jodhpur 2021, and Jodhpur 2022, respectively. The range of LOD and R^2^ values for the SNPs identified by mrMLM and the *P*-value range for the SNPs identified by GAPIT were summarized in Table 2.

**Table 2:** Consistent SNPs across all locations identified for the flowering trait by three or more multi-locus GWAS models

Location	SNP id	LOD/P-value	R^2^ (%)	Contigid	Alleles	Position	Models^*^
Bikaner2021	SNP4779	5.54 to 9.80	5.65 to 11.74	65,980	T/A	8	1,2,3,4,5,6,7
	SNP2396	3.08 to 3.45	4.12 to 6.67	34,123	G/T	179	3,4,5,6,7
	SNP2234	5.74 to 6.90	10.08 to 10.61	31,981	A/C	8	
	SNP3620	4.45 to 6.20	12.10 to 13.83	51,790	A/G	122	2,3,4,7
	SNP5026	0.000331 to 0.000646	-	69,414	C/A	24	4,6,7
	SNP6240	0.000471 to 0.000542	-	90,821	T/C	40	5,6,7
Bikaner2022	SNP858	4.90 to 13.72	7.70 to 16.83	9445	C/T	194	1,2,4,5,7
	SNP6703	5.0 to 13.50	8.24 to 13.31	99,723	C/T	60	1,2,3,4,5,7
	SNP6919	3.32 to 4.17	5.84 to 10.63	104,203	A/G	15	2,3,4,5,6,7
	SNP4472	1.41E-05 to 0.000525	-	62,079	C/T	10	5,6,7
	SNP4735	2.48E-05 to 0.000524	-	65,331	G/A	198	
	SNP9065	4.82E-06 to 0.000951	-	1,416,336	C/T	124	
Jodhpur2019	SNP268	3.90 to 5.93	2.51 to 2.97	1890	A/G	102	1,3,4,5,6,7
	SNP3620	3.26 to 4.51	6.92 to 12.84	51,790	A/G	122	2,3,4,6
	SNP4779	5.56 to 7.96	7.774 to 10.59	65,980	T/A	8	1,2,3,4,5,6,7
	SNP6919	3.43 to 4.32	6.78 to 11.40	104,203	A/G	15	2,3,4,5,6,7
	SNP9076	4.19 to 4.93	7.57 to 9.49	1,436,316	C/T	155	
	SNP4701	4.55 to 5.83	3.00 to 3.74	64,944	GA	98	2,3,4
	SNP5572	6.29E-05 to 0.000578	-	80,466	G/A	48	3,6,7
	SNP1503	7.36E-06 to 9.01E-05	-	19,323	C/A	17	5,6,7
	SNP279	5.00E-05 to 0.000372	-	2196	G/A	277	
Jodhpur2021	SNP4963	3.62 to 5.20	1.96 to 4.02	68,557	A/G	178	1,2,3,4,5,6,7
	SNP3448	3.72 to 5.32	4.19 to 4.95	49,714	C/T	131	2,3,4
	SNP6972	3.10 to 3.52	3.51E-10 to 3.41	105,244	C/A	298	1,3,6
	SNP6349	3.58 to 5.10	4.96 to 8.19	92,715	T/C	197	1,2,4
	SNP3112	3.06 to 3.32	2.06 to 4.52	43,947	G/A	171	2,4,5
	SNP5059	3.07 to 4.16	2.85 to 2.86	70,169	G/A	4	2,3,7
Jodhpur2022	SNP4779	11.21–17.04	10.16 to 18.83	65,980	T/A	8	1,2,3,4,5,6,7
	SNP8592	3.74 to 5.37	1.80 to 6.25	469,510	G/A	80	1,2,3,4,5,7
	SNP4936	3.71 to 3.97	1.84 to 5.64	67,953	C/T	114	1,3,4,5,6,7
	SNP6721	3.05 to 6.53	5.41 to 7.46	100,323	A/T	34	2,4,5,6
	SNP6793	0.000178 to 0.000831	-	102,366	T/A	110	4,5,7
	SNP6919	3.41E-06 to 0.000431	-	104,203	A/G	15	2,5,6
	SNP1371	8.37E-06 to 0.000631	-	17,452	A/G	155	5,6,7

^*^Models: 1: FASTmrEMMA; 2: FASTmrMLM; 3: ISIS EM-BLASSO; 4: mrMLM; 5: Blink; 6: FarmCPU; 7: MLMM

The fitting of the multi-locus GWAS methods was tested using the quantile–quantile (QQ) plots between observed and expected *P*-values of the association that exposed a good fitting for the used methods. Manhattan and QQ plots for days to 50% flowering at Bikaner and Jodhpur (the year 2022) generated by various models in the mrMLM package have been shown in Figs. 6 and 7. For the other two years, these plots are shown in Supplementary Figure S2. Two SNPs (SNP858 at contig 9445 and SNP6703 at contig 99,723) for days to 50% flowering at the Bikaner location (the year 2022) were consistently detected in three models of mrMLM package (FASTmrEMMA, FASTmrMLM, and mrMLM) (Fig. 6A-C). Another consistent SNPs (SNP6919; contig 104,203) with methods FASTmrMLM and mrMLM is depicted in Fig. 6B, C. Similarly, for the Jodhpur location (the year 2022), the top SNPs associated with days to 50% flowering have been depicted by the pink dot on the Manhattan plots of respective methods (Fig. 7A-C). The SNP4779 (contig 65,980) had the highest LOD value was predicted by all methods and shown by pink dots in Fig. 7. Other consistent SNPs such as SNP8592 on contig 469,510, SNP4936 on contig 67,953, SNP6721 at contig 100,323 shown on Manhattan plots for Jodhpur 2022 are summarized in Table 2.

Out of total 34 consistent SNPs detected for days to 50% flowering (Table 2), three SNPs such as SNP4779, SNP3620, and SNP6919 were predicted by all six GWAS methods and were present in more than one location (Table 3). SNP4779 was predicted by all six methods and presented at Bikaner (2021), Jodhpur (2019), and Jodhpur (2022). SNP3620 was predicted by all methods except FasTmrEMMA and Blink and presented at Bikaner (2021) and Jodhpur (2019). SNP6919 was also predicted by all methods except FasTmrEMMA and was present at Bikaner 2022, Jodhpur 2019, and Jodhpur 2022. Based on the distribution across the locations and predicted by multiple methods with high LOD/*P*-value, these SNPs were considered top SNPs.

**Table 3:** Consistent SNPs filtered through different models across both the locations and years

Model	SNP ID	Alleles	Contig	Position	Location
FasTmrEMMA	SNP4779	T/A	65,980	8	B2021, J2019, J2022
FASTmrMLM	SNP3620	A/G	51,790	122	B2021, J2022
	SNP4779	T/A	65,980	8	B2021, J2019 J2022
	SNP6919	A/G	104,203	15	B2022, J2019, J2022
ISIS EM-BLASSO	SNP3620	A/G	51,790	122	B2021, J2019
	SNP4779	T/A	65,980	8	B2021, J2019, J2022
	SNP6919	A/G	104,203	15	B2022, J2019, J2022
mrMLM	SNP3620	A/G	51,790	122	B2021, J2019
	SNP4779	T/A	65,980	8	B2021, J2019, J2022
	SNP6919	A/G	104,203	15	B2022, J2019, J2022
Blink	SNP4779	T/A	65,980	8	B2021, J2019 J2022
	SNP6919	A/G	104,203	15	B2022, J2019, J2022
FarmCPU	SNP3620	A/G	51,790	122	B2021, J2019
	SNP4779	T/A	65,980	8	B2021, J2019 J2022
	SNP6919	A/G	104,203	15	B2022, J2019, J2022
MLMM	SNP3620	A/G	51,790	122	B2021, J2019
	SNP4779	T/A	65,980	8	B2021, J2019, J2022
	SNP6919	A/G	104,203	15	B2022, J2019, J2022

*B* Bikaner, *J* Jodhpur

### Candidate gene analysis and annotation

Candidate genes were investigated for the SNPs (marker-trait associations) significantly associated with days to 50% flowering. A total of 34 SNPs were identified across all environments and in three or more methods. However, out of 34 SNPs, three SNPs (SNP4779, SNP3620, and SNP6919) were present in more than one environment. Thus, essentially, there were only 29 unique SNPs representing 34 associations for flowering traits across environments. These 29 SNPs were subjected to annotation analysis using the BLASTx program. Out of 29 significant unique SNPs, 7 SNPs were found in the vicinity of annotated genes, and the rest were having no hits or were found to be hypothetical (Table 4). The identified annotated genes were involved in a wide variety of functions. These significant candidate genes encoded Hydroquinone glucosyltransferase, LRR receptor-like serine/threonine-protein kinase, Phosphatase and actin regulator, Triplex capsid protein, Actin-related protein 8-like, Cyanogenic beta-glucosidase-like, MLP-like protein 28, and Pectinesterase 3 (Table 4).

**Table 4:** Details of candidate genes and their annotation

SNP	Contig id	Annotation
SNP268	1890	No hit^a^
SNP279	2196	MLP-like protein 28
SNP858	9445	Hypothetical protein LR48
SNP1371	17,452	No hit
SNP1503	19,323	No hit
SNP2234	31,981	Hydroquinone glucosyltransferase-like
SNP2396	34,123	Hypothetical protein VIGAN_11233500
SNP3112	43,947	No hit
SNP3448	49,714	No hit
SNP3620	51,790	Hypothetical protein LR48
SNP4472	62,079	Cyanogenic beta-glucosidase-like
SNP4701	64,944	Pectinesterase 3
SNP4735	65,331	Hypothetical protein LR48
SNP4779	65,980	serine/threonine-protein kinase
SNP4936	67,953	Phosphatase and actin regulator, Triplex capsid protein
SNP4963	68,557	No hit
SNP5026	69,414	Hypothetical protein LR48
SNP5059	70,169	No hit
SNP5572	80,466	No hit
SNP6240	90,821	No hit
SNP6349	92,715	No hit
SNP6703	99,723	Hypothetical protein LR48
SNP6721	100,323	No hit
SNP6793	102,366	No hit
SNP6919	104,203	Actin-related protein 8-like
SNP6972	105,244	Uncharacterized protein LOC106767326
SNP8592	469,510	No hit
SNP9065	1,416,336	No hit
SNP9076	1,436,316	No hit

^a^No hit: Located in intergenic regions

### Allelic effect of reliable SNPs on respective phenotypes

**Fig. 8:**
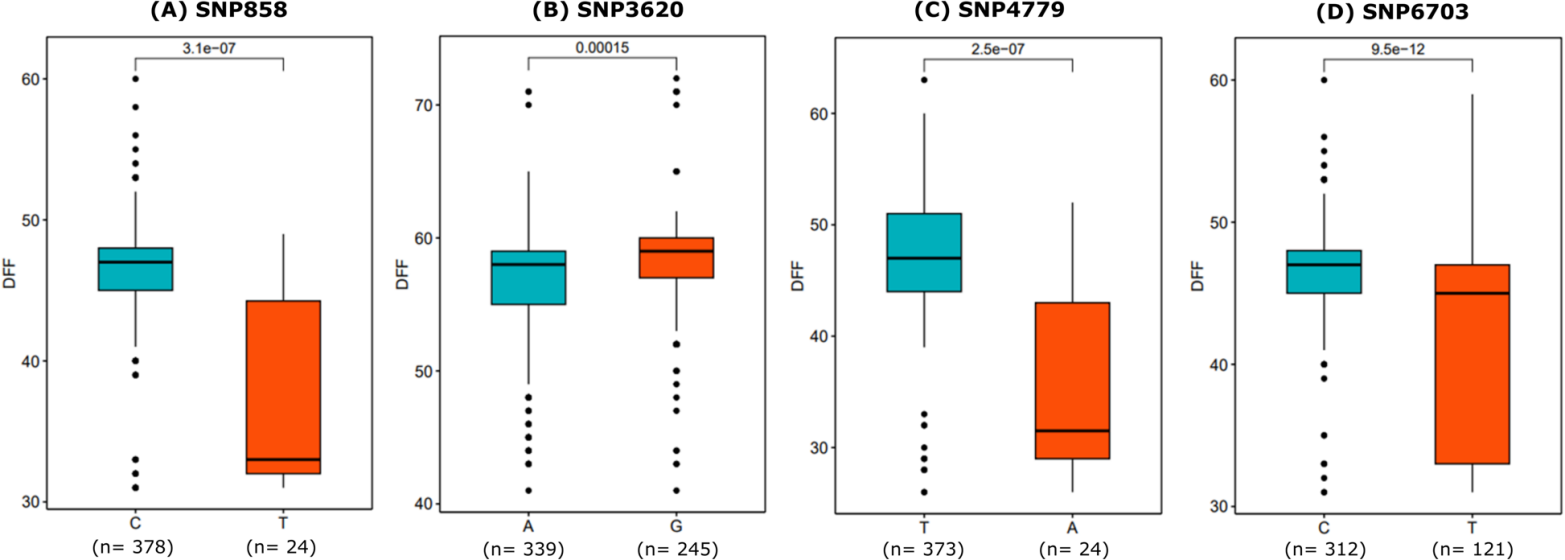
Boxplot for four potential SNPs (**A-D**). Based on allele type, genotypes were categorized into two groups at each locus. The statistical analysis for significant differences between the phenotypes was performed using a t-test (*P* ≤ 0.01). The x-axis shows the two alleles for each SNP and the y-axis shows the phenotypic values for the days to 50% flowering (DFF) trait

Three consistent SNPs (SNP3620, SNP4779, and SNP6919) across the 2 locations (Bikaner and Jodhpur) (Table 3) and SNPs/ genomic regions with R^2^ ≥ 10 were examined for their significant allelic effect on the days to 50% flowering trait. Based on the allele types, the association panel genotypes were divided into two groups to determine whether the mean phenotypes of the two groups were significantly different. We used the t-test to determine the statistically significant difference (*P* value < 0.01) between the two groups of phenotypes. The analysis demonstrated that four SNPs had a significant effect (*P* < 0.01) on the flowering trait (Fig. 8). These significant SNPs reveal that such genomic regions have a potential role in the flowering of moth bean.

## Discussion

Moth bean is amongst the primitive as well as underutilized crop of genus *Vigna.* It demonstrates resilience towards abiotic stress such as heat and drought and is a good source of protein for the sizable population in arid and semi-arid regions of Asia. However, due to a lack of well-characterized genomic resources as well as sequence-based molecular markers, genetic and genomic dissection of moth bean is in its initial stages. The present study uses robust, multiplexed, high-throughput and low-cost GBS technique to discover genome-wide high-quality SNPs in a diverse panel of moth bean. To the best of our knowledge, the present study is the first report that uses the genome-wide association to map genomic regions related to any trait in this crop.

A total of 9078 filtered SNPs and a sub-set of 428 accessions were used for population structure analysis. These accessions were grouped into two subgroups based on LnP (D) using the admixture model. For a better representation of genetic variability within the population, individuals within populations were categorized as pure and admixtures (Fig. 3A). Majority of accession used in this study were collected from Rajasthan (160) and Gujarat (56). Population structure analysis resulted in the categorization of 104 accessions from Rajasthan under subpopulation 2 along with only 13 accessions in subpopulation 1. Contrastingly, the majority of accessions from Gujarat were confined to subpopulation 1 (25 accessions) while only 11 accessions were grouped in subpopulation 2 and the rest were admixtures (20 accessions). Genetic variation within the population analyzed through AMOVA and PCoA also represented similar results. The genetic variation among the population as well as among individuals was significantly lower than that found within the individual (Fig. 4A). This might be due to the self-pollinating nature of moth bean that show low genetic heterogeneity representing high genetic relatedness [32]. However, the finding in the present study showed genetic distinctness between the accessions collected from Rajasthan and Gujarat. Because large number of distinct accessions found in these two regions than in any other regions of the Indian subcontinent further support the view that the origin of moth bean is confined to regions of Rajasthan and Gujarat. Population structure analysis in closely related species of moth bean such as black gram and mung bean showed population distribution into four subpopulations [37, 38]. PCA analysis and the phylogenetic tree using the NJ method also showed that 428 accessions were grouped into two clusters. Therefore, the PCA and phylogenetic results were in agreement with the result of admixture-based model that represented the moth bean genotypes were grouped into two clusters. Also, the grouping pattern of the population was mostly dependent on geographic origin. Similar agreement of results was observed in mung bean and moth bean [37, 38]. Population distribution of black gram and mung bean in four different subpopulations could be due the fact that center of origin and domestication of both the species is spread across southeast Asia [39]; while the distribution of the moth bean population into two subgroups.

Flowering time is a key phenological trait that governs plant growth, plant height, and grain quality. Early flowering varieties generally mature earlier that save input costs and it also helps the plant to escape abiotic stress such as drought and heat [40, 41]. The moth bean diversity panel used in the present study showed large variation for days to 50% flowering trait across environments. Thus, in order to map the genomic regions for flowering trait the selected moth bean diversity panel was evaluated at five environments distributed over three years i.e., 2020, 2021, 2022. Twenty-nine significant unique SNPs were predicted by seven multi-locus models for different locations (Table 2). Among these SNPs (genomic regions) such as SNP4779, SNP3620, and SNP6919 were detected by all the seven GWAS models suggesting these to be the important genomic regions responsible for the flowering trait. These SNPs can be validated further using a functional genomics approach for their utilization in a breeding program. Contigs bearing these significant SNPs were used for annotation by performing the BLASTx with *Vigna* genomes along with model plants. Seven contigs with an e-value below 0.05 showed significant annotation with the genome of other closely related *Vigna Spp.* These were found to show significant association with the flowering mechanisms. The receptor-like serine/threonine-protein kinases belong to the largest group of family of receptor-like kinases (RLKs) and receptor-like proteins (RLPs) which are actively involved in sensing cell’s external and internal signals [42]. These proteins, therefore, modulate specific cellular responses involving plant innate immunity, growth, and cell differentiation [43, 44]. Lin et al. showed tyrosine phosphorylation of BIK1 (Botrytis-induced kinase 1), a receptor like-cytoplasmic kinases involved in the immunity of plants through pattern-triggered immune (PTI) signaling [45]. The *BIK1* mutant has been reported to show defects at the later developmental stages with early flowering [46]. The function of hydroquinone glucosyltransferase has been described by Arend et al. using plant cell suspension cultures of *Rauvolfia serpentine* [47]. The study demonstrated the conversion of hydroquinone to arbutin (*O*-ß-D-glucoside of hydroquinone) with the help of the enzyme hydroquinone:*O*-glucosyltransferase. Glycosyltransferases (GTs) are enzymes that glycosylate plant compounds that in turn are directly or indirectly involved in the production of plant secondary compounds. A study involving *Arabidopsis* glycosyltransferase (*UGT87A2*) depicted regulation of flowering time involving flowering repressor gene *FLOWERING LOCUS C* [48]. The study further showed that mutant of glycosyltransferase (*UGT87A2*) extended the flowering time in *Arabidopsis* for both long and short days [48]. The annotation also positioned contig id 31,981 and 62,079 on chromosome 5 of *V. unguiculata* (Table 4). Lo et al. reported two major QTLs related to flowering time on Chromosome Vu5 and Vu9 of *V. unguiculata* [49]. However, the QTL found on Vu5 showed only 20% of PVE (Phenotypic variance explained) compared to that of Vu9 (79.3%). Contig id 2196 (SNP279) annotated as MLP (Major latex protein)-like protein. The gene of MPL was first identified in opium poppy latex and its orthologs that are found in different plants were called as MPL-like protein [50, 51]. The role of MPL induced by cis-cinnamic acid in promoting vegetative growth and delayed flowering time in *Arabidopsis* has been reported [52]. Contig id 64,944 (SNP4701), annotated as pectin esterase demethylate pectin in plants to yield pectate which is involved in plant cell wall modification. Albani et al. reported genomic clone (Bp19) of *Brassica napus* contained a gene annotated as pectin esterase [53] which showed expression throughout the developmental stages of pollen. Higher expression of this gene was observed in the early stages of pollen development. However, the expression decreased at the mature pollen stage. The study further concluded the direct involvement of the pectin esterase gene in pollen tube germination. Mu et al. also reported the expression of the putative pectin esterase (*PPE1*) gene in mature pollen and germinated pollen tubes [54]. The study further reported increase in expression of *PPE 1* gene in the subsequent developmental stage of anther which was highly positively correlated with the size of the flower. Contig id 104,203 (SNP6919) was annotated as Actin-related protein 8 like. It belongs to actin-related protein (ARP) family that are represented proteins having similar identity between 17 and 60% with conventional actins. In accordance to the subcellular localization, it is categorized either as cytoplasmic (ARP1, 2, 3, and 10) or nuclear (ARP4, 5, 6, 7, 8, and 9) [55]. The function of ARP8 has not been fully understood in plants [56]; however, the role of its homologue ARP6 and APR4 in controlling flowering timing and floral development has been well studied in *Arabidopsis* [55, 57]. Many contigs were having no hits or were found to having uncharacterized coding genes (Table 4).

Chromosomal localization of significant SNPs (9078) on the genomes of closely related *Vigna* species such as *V. angularis*, *V. mungo*, *V. radiata*, *V. umbellata*, *V. unguiculata* was performed for deducing genome level relatedness among these species. It resulted in the localization of 3721 (40.99%), 6155 (76.80%), 3807 (41.94%), 3681 (40.55%), and 1391 (15.32%) with the genome of *V. angularis*, *V. mungo*, *V. radiata*, *V. umbellata* and *V. unguiculata,* respectively. The result therefore clearly demonstrates a high level of genomic similarity of the moth bean genome with that of *V. mungo* followed by *V. radiata* while the lowest similarity was observed to be with genome of *V. ungiculata*. The result is line with the phylogenetic positioning of *Vigna* species reported by Takahashi et al. [58] that showed phylogenetic similarity of *V. aconitifolia* with *V. radiata* followed by *V. umbellta* and *V. unguiculata* using DNA sequences of nuclear rDNA-ITS and chloroplast *atpB-rbcL* spacer regions. Genomic synteny of moth bean performed by Yundaeng et al. [7] compared common SSRs used for the construction of moth bean linkage map with that of closely related *Vigna* species such as azuki bean, rice bean, mungbean, black gram and yardlong bean. The study demonstrated a high level of moth bean genomic synteny with *Vigna* species, especially with azuki bean. Nearly 76.2% of azuki bean SSRs were found to be amplified while accessing parental polymorphism within moth bean parents.

## Conclusion

The characterization of the genetic diversity of moth bean accessions using GBS showed that accessions from the northwest part of India were highly diverse and this region may represent the center of diversity and domestication. GWAS analysis for the flowering trait in moth bean using seven multi-locus-based models identified a total of 29 significant SNPs from five different flowering datasets. These significant SNPs were identified by three or more methods. A total of four major genomic regions showed a significant effect on the flowering trait. Further, the sequence-based localization analysis revealed that *V. mungo* was the closest species to moth bean among all *Vigna* species. The potential candidate genes/ SNPs identified in this study may be further validated for their role in determining days to 50% flowering and used for the development of markers that may be exploited in the marker-assisted program for breeding early-maturity moth bean varieties.

## Materials and methods

### Plant materials

**Fig. 9:**
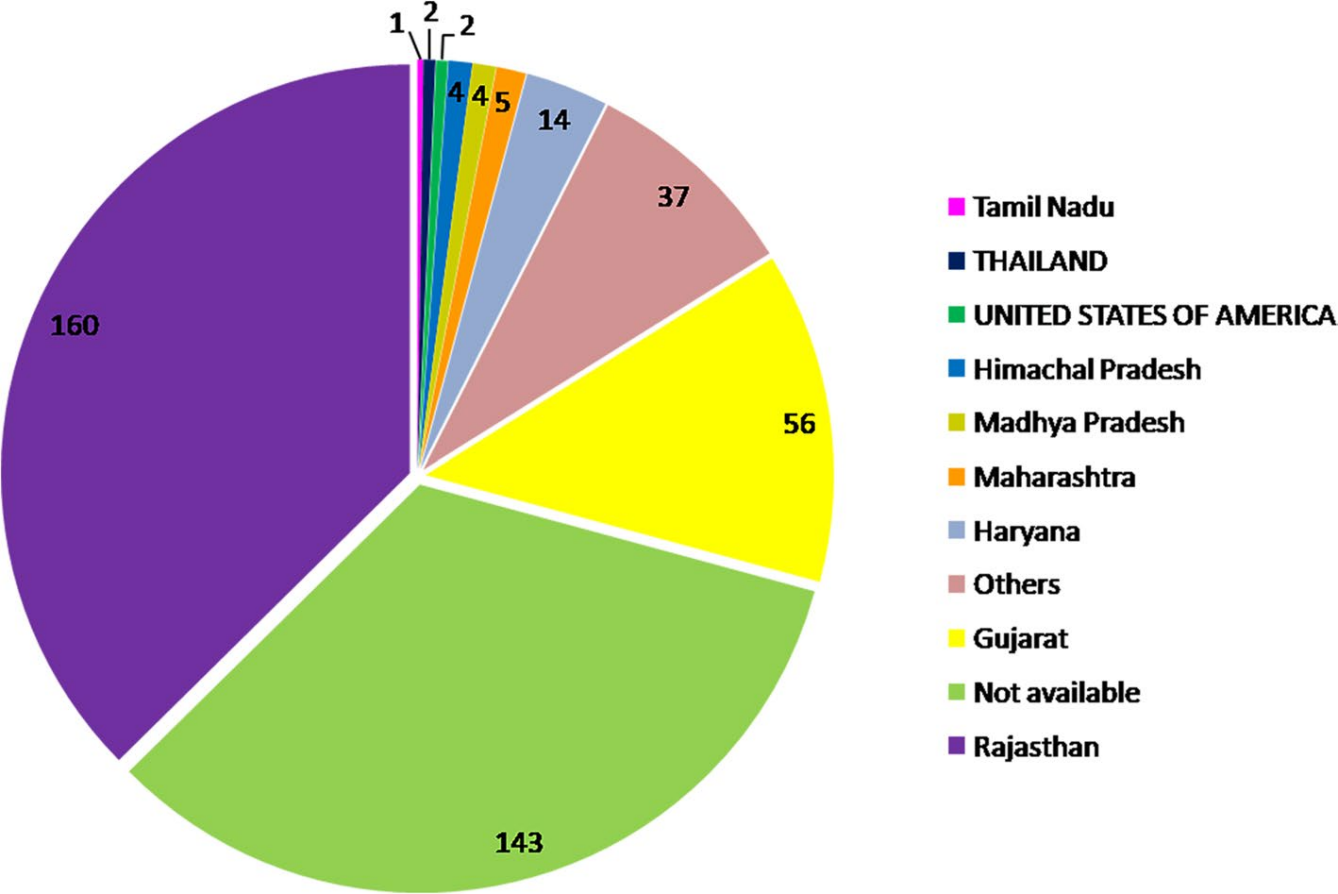
Pie chart representing ecological distribution of 428 moth bean accessions based on their site of collection. These accessions are available in the National gene bank (NGB) of India at ICAR-NBPGR. The maximum number of accessions is confined to Rajasthan

A total of 428 moth bean accessions were analyzed in the present study. The seeds of all accessions were collected from the genebank of ICAR-NBPGR, New Delhi. These 428 moth bean accessions were collected from various locations such as 160 from Rajasthan, 56 from Gujarat, 14 from Haryana, 4 from Madhya Pradesh, 5 from Maharashtra, 4 from Himachal Pradesh, 1 from Tamil Nadu, 2 from Thailand and 2 from United States of America (USA) (Fig. 9). Details of all the 428 moth bean accessions are presented in Supplementary Table S5.

### Experimental design and phenotypic collection

**Fig. 10:**
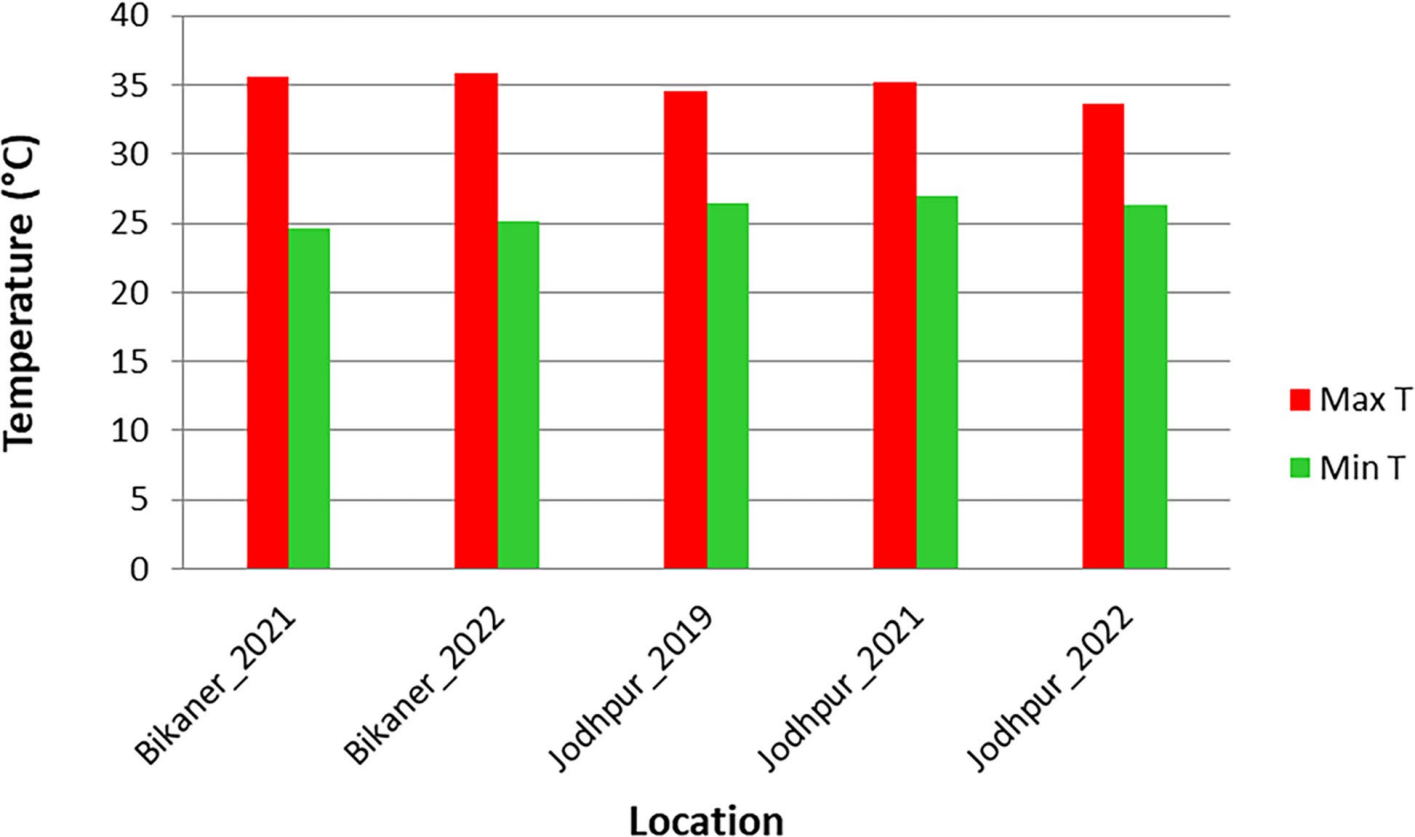
The graph showing average maximum (Max T) and minimum (Min T) daily temperature during the analysis of days to 50% flowering trait of 428 moth bean accessions across sampling locations of Bikaner and Jodhpur in their respective years

All materials were planted in two different locations. The first location was Bikaner (28°1′37.60′′ N latitude and 73°18′7.76′′ E longitude) during the growing session in 2021 and 2022. The second location was Jodhpur (26°15′ 49.91′′ N latitude and 73° 0′ 32.25′′ E longitude) during the growing session in 2019, 2021 and 2022. During the flowering period, the average maximum and minimum temperature was 35.61 °C and 24.68 °C in 2021 and 35.88 °C and 25.2 °C in 2022 at Bikaner respectively. The average maximum and minimum temperatures at Jodhpur were 34.6 °C and 26.4 °C in 2019, 35.2 °C and 26.9 °C in 2021, and 33.6 °C and 26.3 °C in 2022 (Fig. 10). The experiments were laid following Augmented Block Design with five checks (CZM-2, RMO-40, RMO-225, RMO-257, and RMO-435). Each entry was shown in 2 rows of 2 m in length. Checks were repeated 2 times in each block. The day of flowering (days to 50% flowering) was considered from the day of seeding to the first time 50% of the plants of a given accession flowered. The day of flowering was recorded every 2 days.

### DNA extraction, GBS and SNP calling

Genomic DNA was isolated from the leaf of two-week-old moth bean seedlings using CTAB (cetyl trimethyl ammonium bromide) method [59] and treated with RNase to remove RNA contamination. The integrity of DNA samples was tested on 0.8% agarose gel and concentration was determined using NanoDrop1000 (Thermo Scientific). Genotyping was performed using the GBS approach. The DNA sample of each genotype was digested using *Mse1* and *HindIII_EcoR1* restriction enzymes and resulting fragments were ligated with two barcoded adapters as well as a universal sequencing adapter. The library was prepared by pooling segments of desired fragment size selected after gel electrophoresis. The library was quantified on Bioanalyzer using high sensitivity DNA kit (Agilent Technologies) and sequenced on the Illumina HiSeq2500 platform. The raw sequence files were parsed based on the barcode, low-quality reads were removed and data was analyzed using ‘Stacks’ software [60]. SNPs were called de novo using Stacks pipeline due to the unavailability of moth bean reference genome sequence [61]. All SNPs with missing data >20% and MAF < 5% were removed.

### Analysis of population structure, PCA, phylogenetic tree and AMOVA

Population structure was estimated using Bayesian model-based approach implemented through the STRUCTURE program version 2.2 [62]. The putative number of a subpopulation (k) in the range of k = 1 to 5 was estimated using a burn-in of 50,000, number of iterations 10 followed by 20,000 Monte Carlo Markov Chain (MCMC). The optimum subpopulation number (k) was estimated using an ad hoc statistic ∆K with the help of STRUCTURE HARVESTER [63]. PCA was calculated using PLINK (version 1.9) and the plot was prepared in ‘R’ software package using SNP data. The dendrogram was created using TASSEL (v4.0) [64] with the Neighbour-joining (NJ) method and the tree was plotted using iTOL [65] to analyze the genetic stratification in the moth bean subset. The AMOVA and PCoA were performed using GenAlEx 6.5 [66].

### Genome-wide association studies

GWAS was carried out using various multi-locus methods such as mrMLM [67], FASTmrMLM [68], FASTmrEMMA [69] and ISIS EM-BLASSO [70] implemented in mrMLM package v4.0 of ‘R’ (https://cran.r-project.org/package=mrMLM) and MLMM [71], BLINK [72] and FarmCPU [73] implemented in GAPIT [74] R package. All the parameters were set by default for the analysis. The multi-locus methods broadly function on the same principle but differ in statistical power and accuracy for estimating the marker effects. The mrMLM package v4.0 implemented in ‘R’ was used to generate the Manhattan and QQ plots [75]. SNPs associated with the traits were identified based on LOD at the threshold of ≥ 3 and *P*-value. SNPs detected in three or more methods were considered as significant SNPs reliably associated with flowering trait.

### Chromosomal Localization of moth bean SNPs

The flanking sequences (100 bp) from each SNP’s position for all 9078 SNPs were used to search against the genome of other *Vigna* species. The standalone BLASTn program was performed with the genome of *Vigna angularis* (adzuki bean), *V mungo* (black gram), *Vigna radiata* (mung bean), *V. umbellata* (rice bean), and *Vigna unguiculata* (cowpea). The blast parameters were set as, Best hit score = 0.05, and Best hit overhang = 0.1. The top hits based on e-value and identity > 77% were selected as the final hits. The top hits and their respective coordinates were used to plot the circus map using the python package pycircos 1.0.2 (https://pypi.org/project/pycircos/). Chromosomal mapping of SNP markers was performed using the web tool MG2C (http://mg2c.iask.in/mg2c_v2.1/) to represent their physical position on *V. mungo* chromosomes [76].

### Candidate gene discovery and annotation

The significantly associated SNPs with flowering trait were searched for the orthologous sequences in genomes of other *Vigna* species as well as in other model crops. The putative candidate genes were identified using the BLASTx program from NCBI-BLAST (https://blast.ncbi.nlm.nih.gov/Blast.cgi) with default parameters. Functional annotations of each putative candidate gene were also investigated using *Vigna* species along with the other model crops.

## Supplementary Information

**Additional file 1: Supplementary Figure S1.** Localization of moth bean SNPs (6155 SNPs) on 11 V. mungo chromosomes.

**Additional file 2: Supplementary Figure S2.** Manhattan and QQ plots generated by the total used model for all environments.

**Additional file 3: Supplementary Table S1.** List of common early flowering accessions across five different environments.

**Additional file 4: Supplementary Table S2.** Distribution of accessions according to population structure and phylogenetic relationship analyses.

**Additional file 5: Supplementary Table S3.** Blast analyses for flanking sequence of all SNPs with other Vigna species such as V. angularis, V. mungo, V. radiata, V. umbellate, and V. unguiculata, representing their genomic association.

**Additional file 6: Supplementary Table S4.** All significant SNPs identified by each models across the all environment.

**Additional file 7: Supplementary Table S5.** All moth bean accessions used in this study along with their distributed location.

## Data Availability

The data presented in this study are submitted to the Sequence Read Archive of the National Center for Biotechnology Information (NCBI) under BioProject: PRJNA922325.

## Abbreviations

*V. aconitifolia*: *Vigna aconitifolia*
GWAS: Genome-wide association study
SNP: Single nucleotide polymorphisms
QTL: Quantitative trait locus
GBS: Genotyping by sequencing
PCA: Principal component analysis
AMOVA: Analysis of molecular variance
PCoA: Principal coordinates analysis
LOD: Logarithm of odds
