# Supplementary figures and images for: Genetic diversity, population structure, and genome-wide association study for the flowering trait in a diverse panel of 428 moth bean (Vigna aconitifolia) accessions using genotyping by sequencing
Source: BMC Plant Biol. 2023 Apr 29;23:228. doi: 10.1186/s12870-023-04215-w (PMC10148550; doi:10.1186/s12870-023-04215-w)

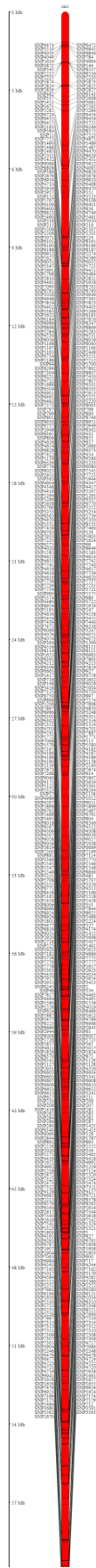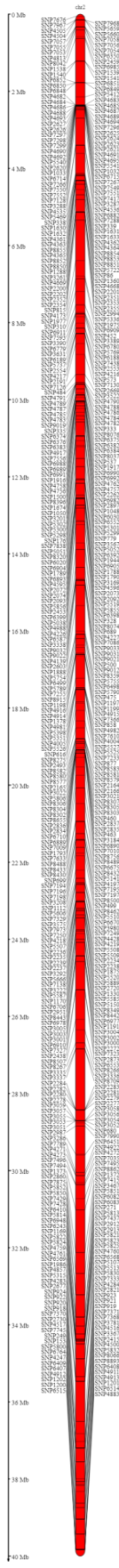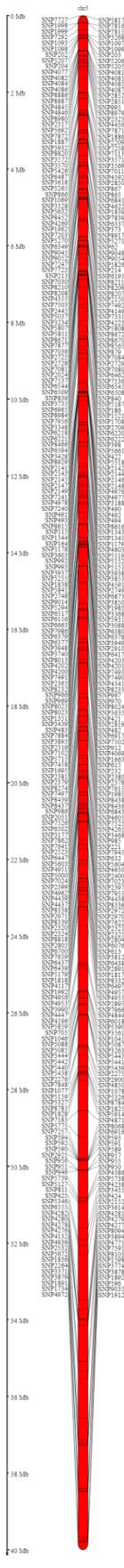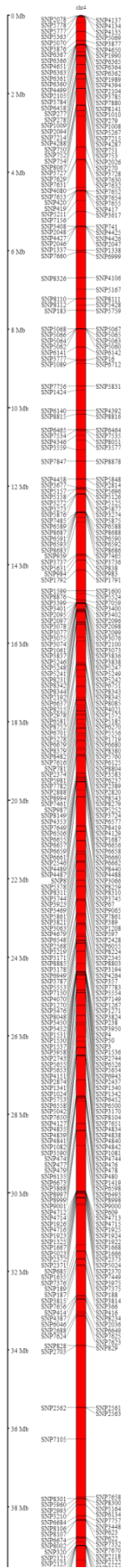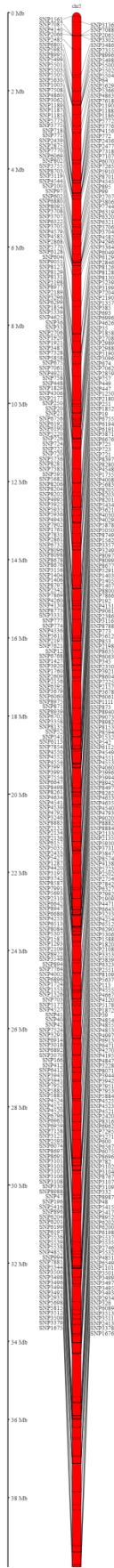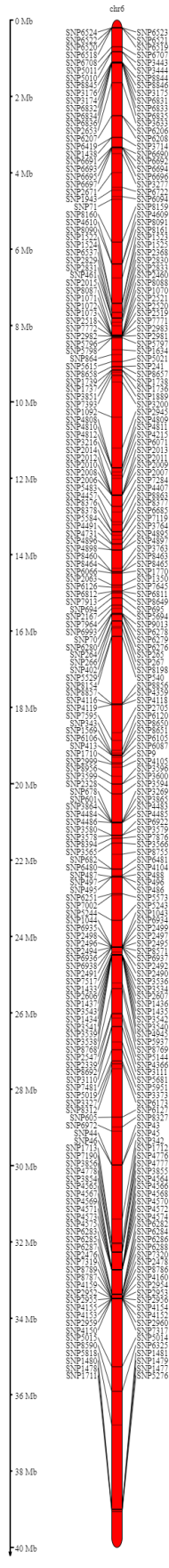

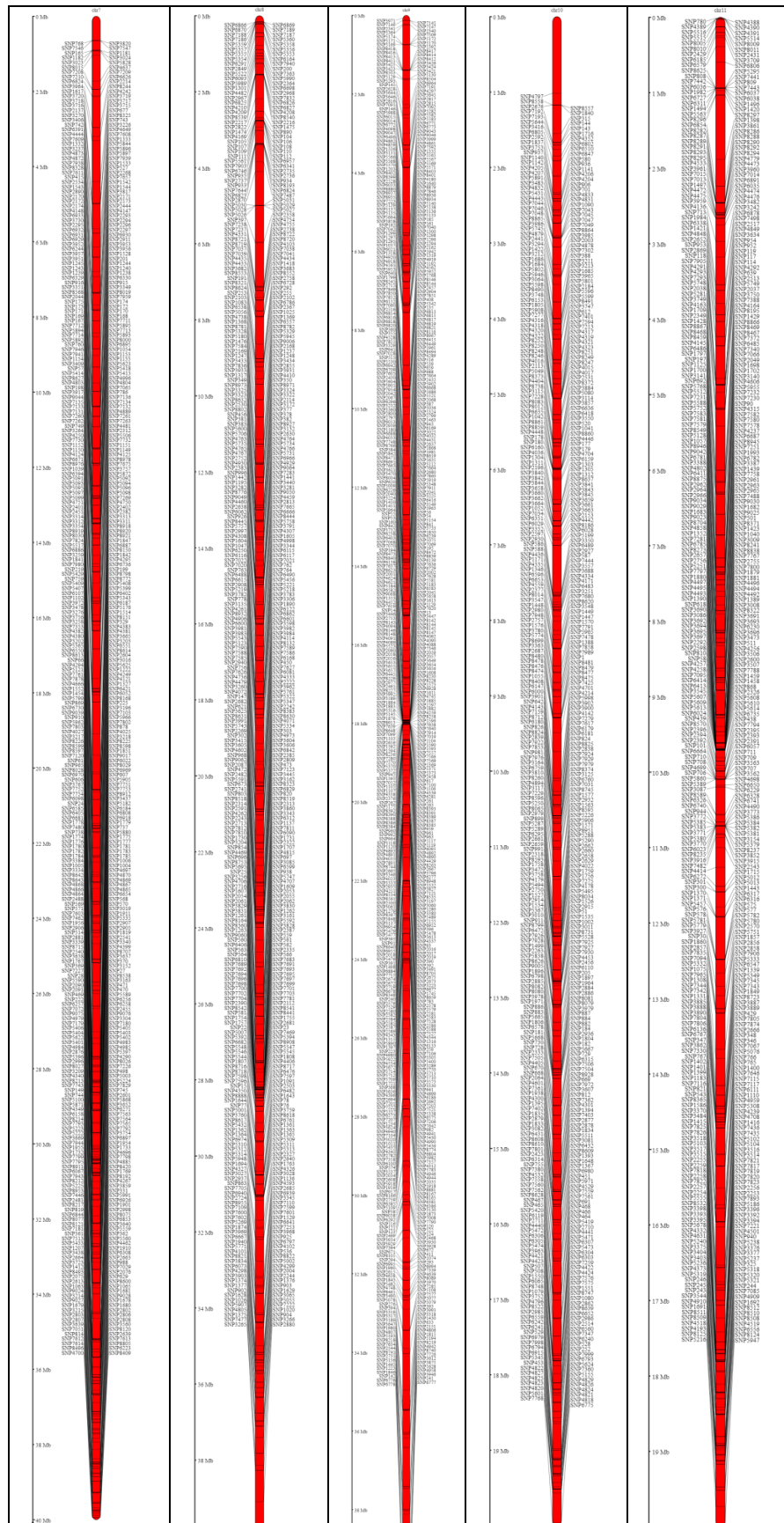

**Supplementary Figure S1:** Localization of moth bean SNPs (6155 SNPs) on 11 *V. mungo* chromosomes.

Supplement: Supplementary file 1 — Additional file 1: Supplementary Figure S1. Localization of moth bean SNPs (6155 SNPs) on 11 V. mungo chromosomes. [file 12870_2023_4215_MOESM1_ESM.pdf]
