# Supplementary material for: Genetic diversity, population structure, and genome-wide association study for the flowering trait in a diverse panel of 428 moth bean (Vigna aconitifolia) accessions using genotyping by sequencing
Source: BMC Plant Biol. 2023 Apr 29;23:228. doi: 10.1186/s12870-023-04215-w (PMC10148550; doi:10.1186/s12870-023-04215-w)
Supplement: Supplementary file 2 — Additional file 2: Supplementary Figure S2. Manhattan and QQ plots generated by the total used model for all environments. [file 12870_2023_4215_MOESM2_ESM.pdf]

1. Bikaner 2021

FASTAmrEMMA

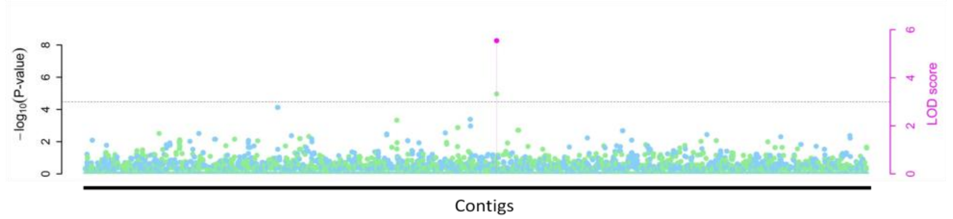

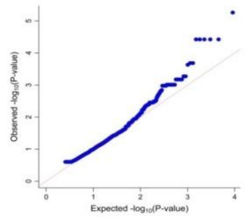

FASTmrMLM

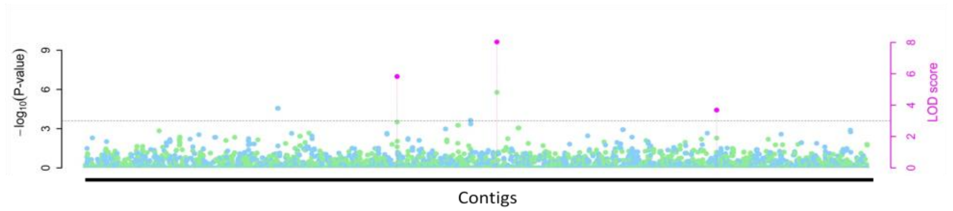

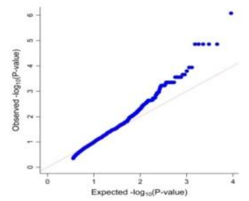

mrMLM

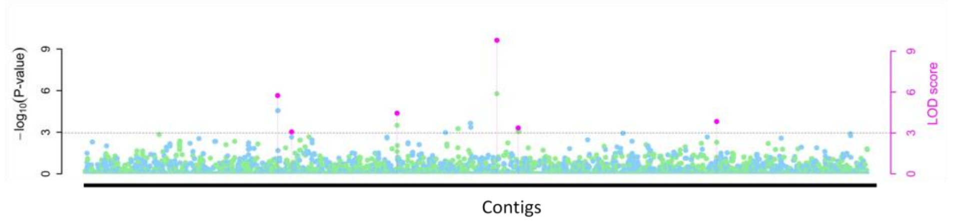

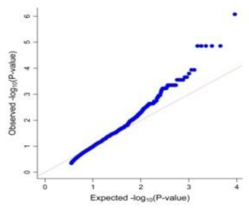

Blink

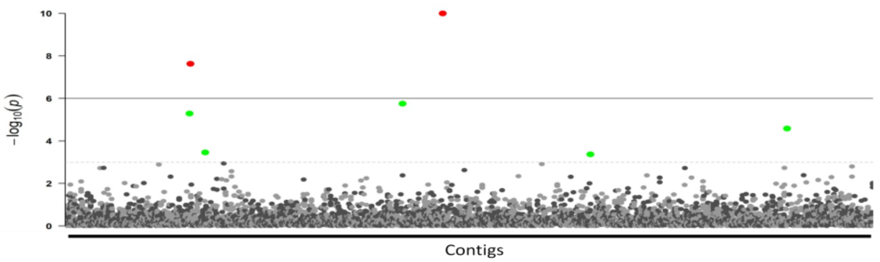

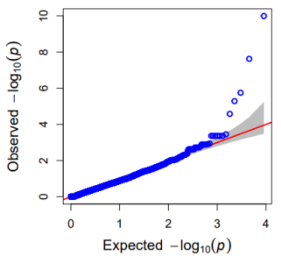

FarmCPU

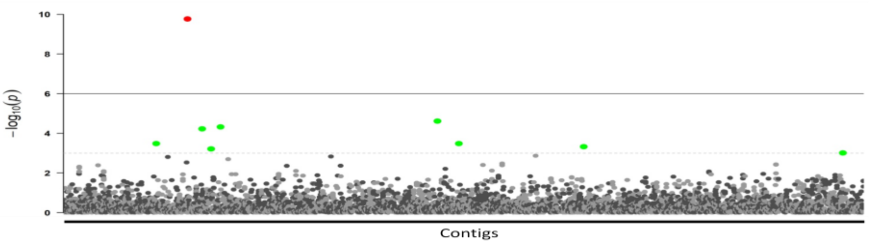

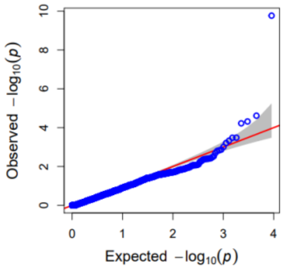

MLMM

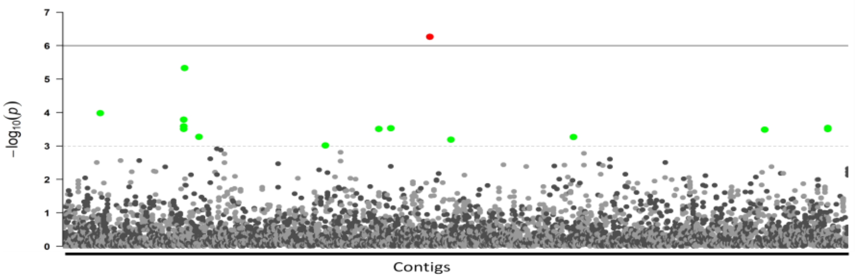

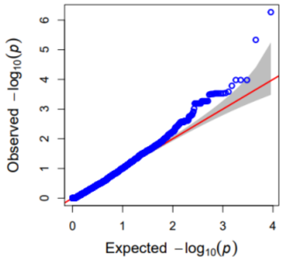

2. Bikaner 2022

<table>
<tr><td>Blink
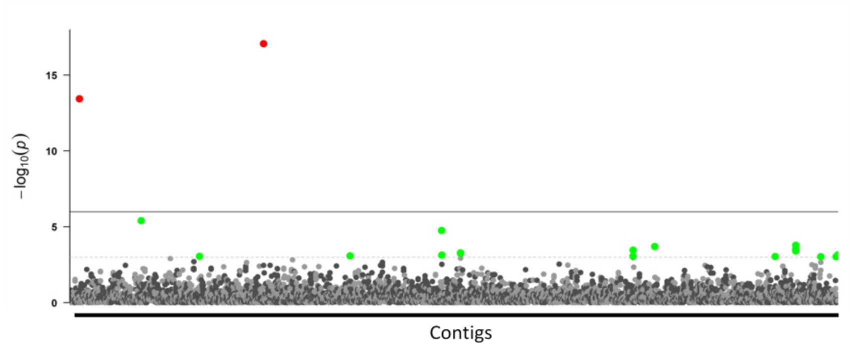
</td><td>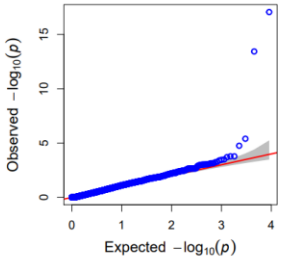
</td></tr>
<tr><td>FormCPU
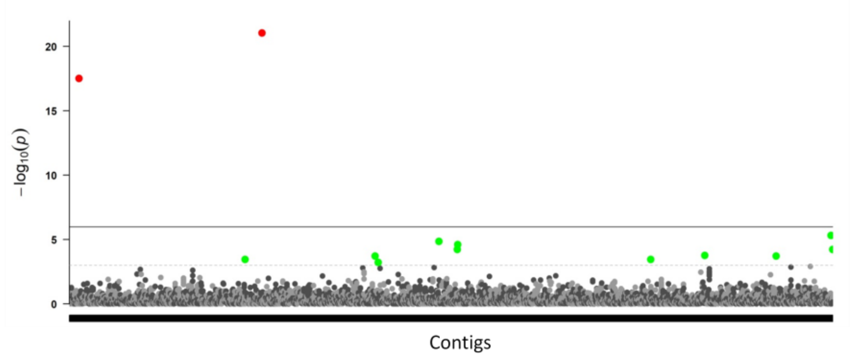
</td><td>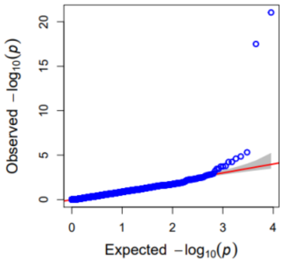
</td></tr>
<tr><td>MLMM
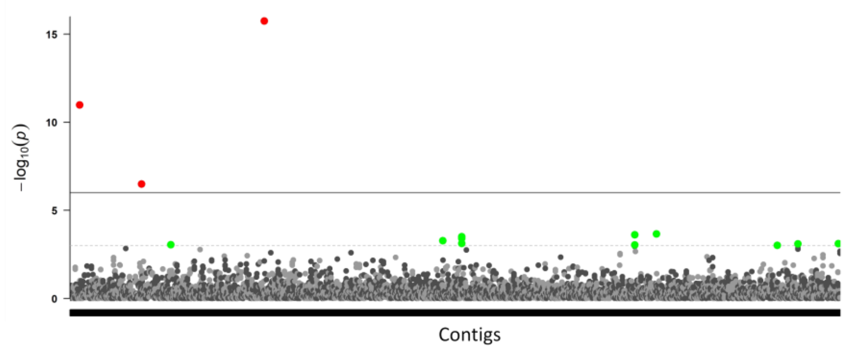
</td><td>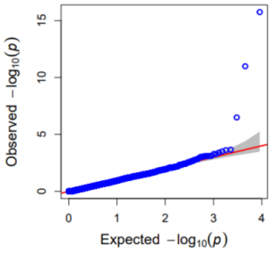
</td></tr>
</table>

3. Jodhpur 2019

FASTmrEMMA

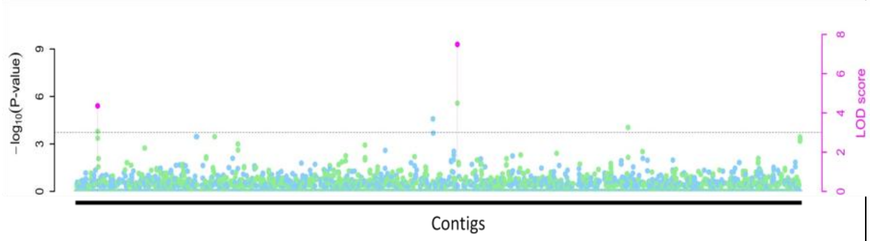

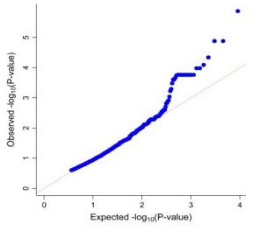

FASTmrMLM

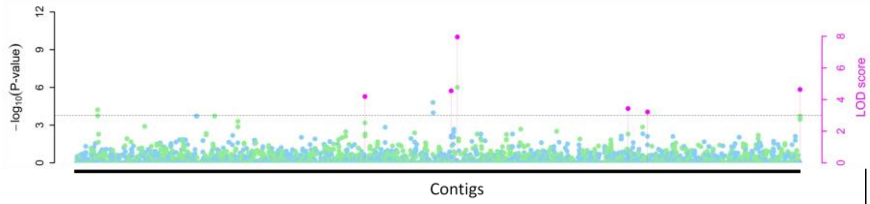

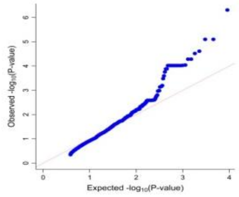

mrMLM

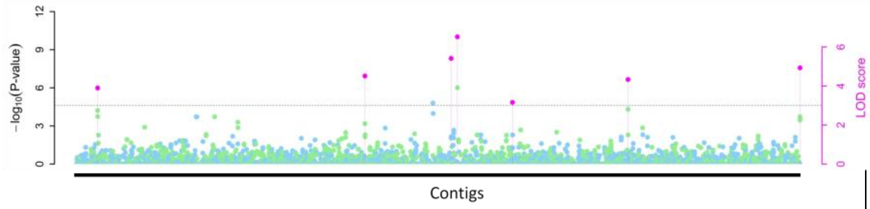

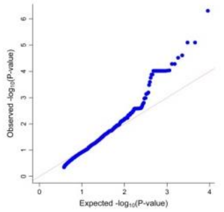

Blink

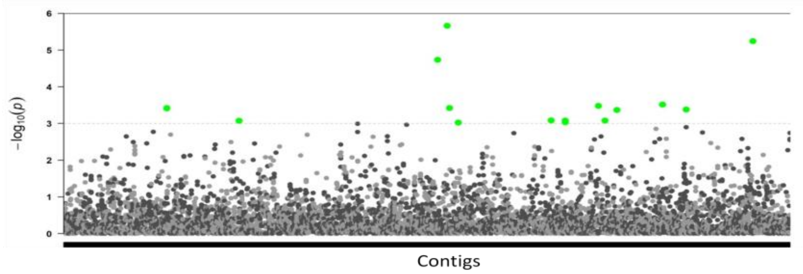

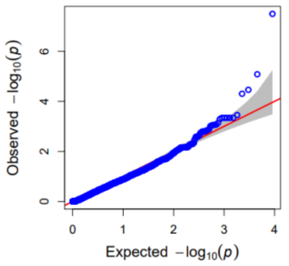

FarmCPU

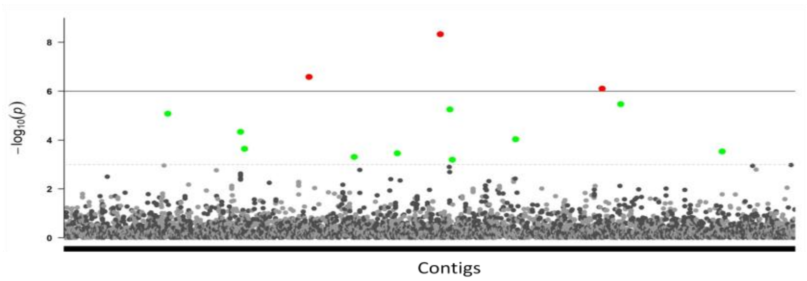

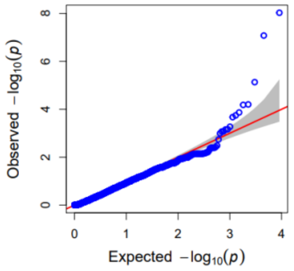

MLMM

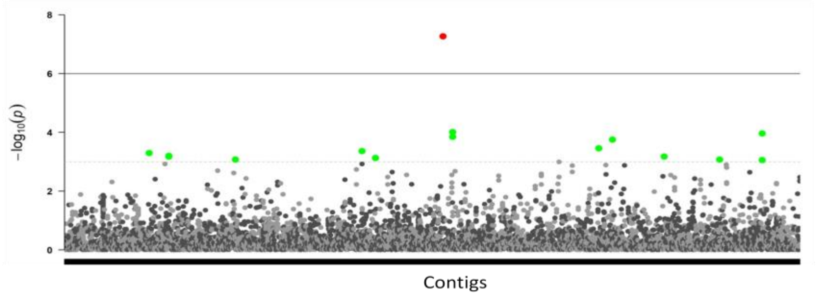

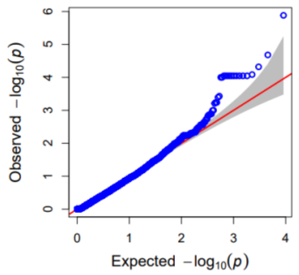

4. Jodhpur 2021

FASTmrEMMA

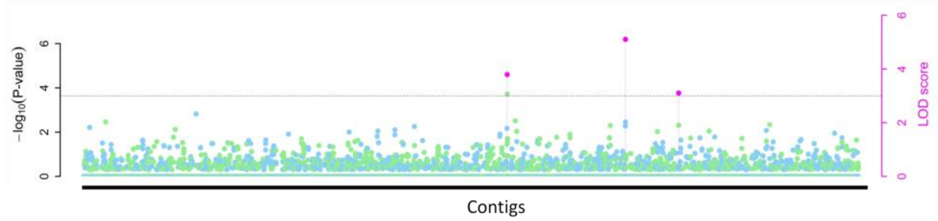

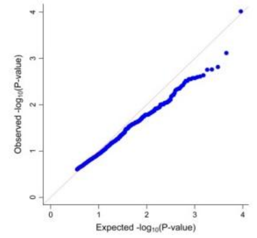

FASTmrMLM

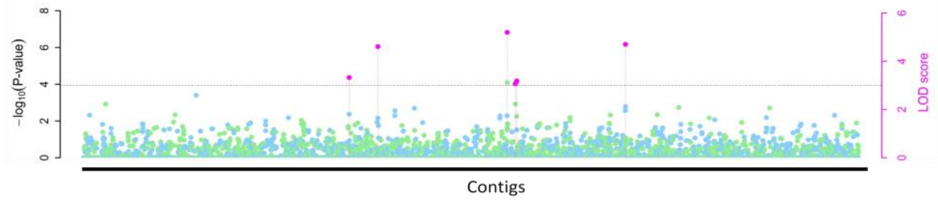

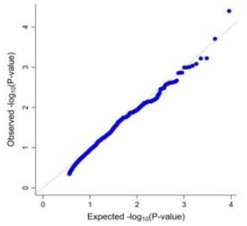

mrMLM

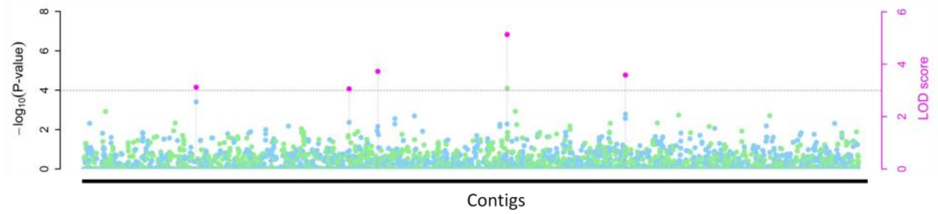

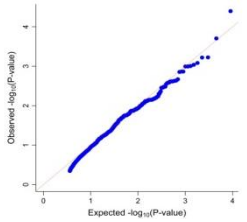

Blink

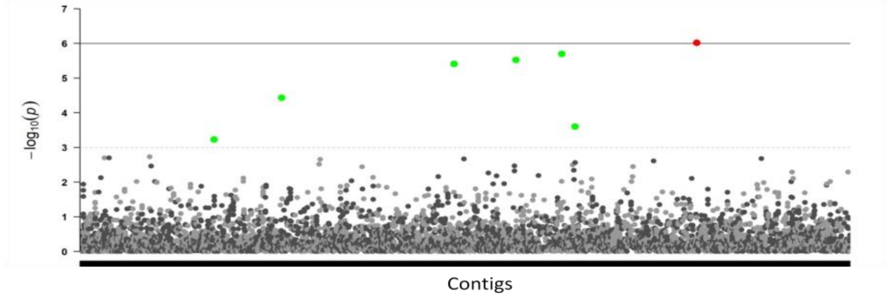

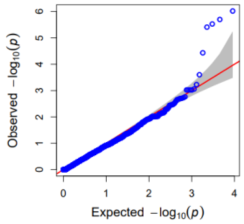

FarmCPU

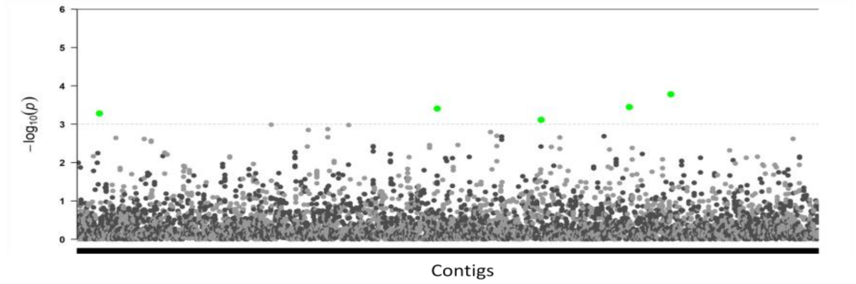

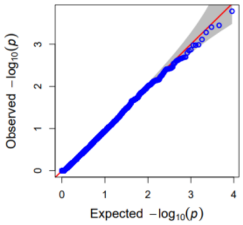

MLMM

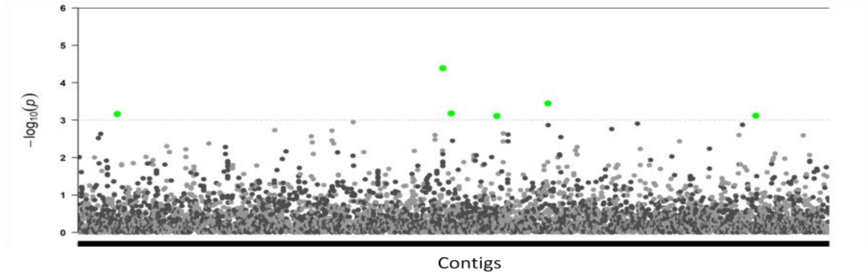

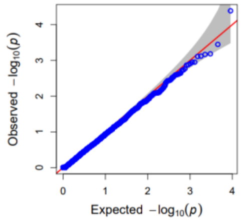

5. Jodhpur 2022

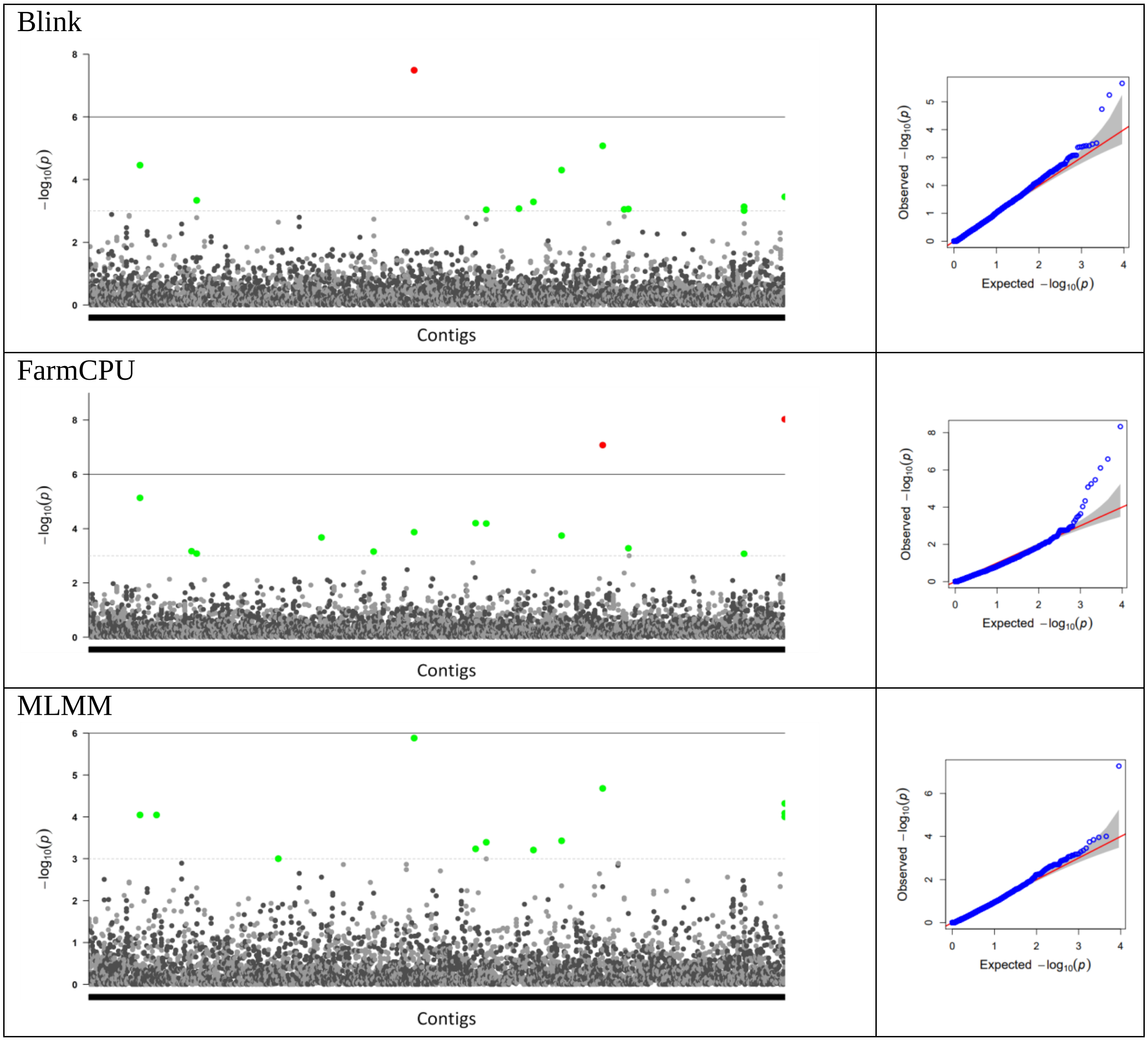

Supplementary Figure S2: Manhattan and QQ plots generated by the total used model for all environments.
