# Supplementary material for: Genetic diversity, population structure, and genome-wide association study for the flowering trait in a diverse panel of 428 moth bean (Vigna aconitifolia) accessions using genotyping by sequencing
Source: BMC Plant Biol. 2023 Apr 29;23:228. doi: 10.1186/s12870-023-04215-w (PMC10148550; doi:10.1186/s12870-023-04215-w)
Supplement: Supplementary file 3 — Additional file 3: Supplementary Table S1. List of common early flowering accessions across five different environments. [file 12870_2023_4215_MOESM3_ESM.docx]

**Supplementary Table S1:** List of common early flowering accessions across five different environments.

|  | **Days to 50% flowering** | | | | | |
| --- | --- | --- | --- | --- | --- | --- |
| **Accessions** | **Jodhpur 2019** | **Jodhpur 2021** | **Jodhpur 2022** | **Bikaner 2021** | **Bikaner 2022** | **Average** |
| IC28155 | 30 | 32 | 26 | 44 | 31 | 32.6 |
| IC140751 | 33 | 33 | 26 | 43 | 31 | 33.2 |
| IC472280 | 32 | 31 | 29 | 46 | 31 | 33.8 |
| IC28156 | 31 | 34 | 28 | 47 | 31 | 34.2 |
| IC415155 | 36 | 34 | 28 | 42 | 31 | 34.2 |
| IC472281 | 32 | 36 | 26 | 45 | 33 | 34.4 |
| IC472279 | 34 | 35 | 29 | 44 | 31 | 34.6 |
| IC28147 | 32 | 38 | 28 | 45 | 31 | 34.8 |
| IC372717 | 35 | 34 | 30 | 44 | 32 | 35 |
| IC8891-1 | 33 | 35 | 32 | 43 | 33 | 35.2 |
| IC415116 | 32 | 37 | 29 | 48 | 31 | 35.4 |
| IC402287 | 36 | 35 | 29 | 46 | 31 | 35.4 |
| IC617820 | 33 | 36 | 28 | 49 | 32 | 35.6 |
| IC372711 | 36 | 35 | 29 | 45 | 33 | 35.6 |
| IC36580 | 33 | 37 | 30 | 46 | 33 | 35.8 |
| IC415152 | 36 | 40 | 27 | 43 | 33 | 35.8 |
| IC623412 | 33 | 39 | 31 | 46 | 31 | 36 |
| IC402289 | 40 | 37 | 26 | 45 | 32 | 36 |
| IC415132 | 34 | 38 | 30 | 45 | 34 | 36.2 |
| IC440239 | 33 | 40 | 29 | 50 | 32 | 36.8 |
| IC415136 | 38 | 34 | 32 | 45 | 35 | 36.8 |
| IC36581 | 38 | 39 | 28 | 48 | 32 | 37 |
| IC8849 | 40 | 36 | 32 | 44 | 33 | 37 |
| IC617784 | 32 | 39 | 32 | 50 | 33 | 37.2 |
| IC415139 | 36 | 39 | 29 | 50 | 33 | 37.4 |
| IC396881 | 40 | 39 | 32 | 47 | 31 | 37.8 |
